# Molecular Cloning and Functional Characterization of *CpMYC2* and *CpBHLH13* Transcription Factors from Wintersweet (*Chimonanthus praecox* L.)

**DOI:** 10.3390/plants9060785

**Published:** 2020-06-23

**Authors:** Muhammad Zeshan Aslam, Xiang Lin, Xiang Li, Nan Yang, Longqing Chen

**Affiliations:** 1 Key Laboratory of Horticultural Plant Biology (Ministry of Education), College of Horticulture and Forestry Science, Huazhong Agricultural University, Wuhan 430070, China; zee125@webmail.hzau.edu.cn (M.Z.A.); xianglin@mail.hzau.edu.cn (X.L.); lixiang@webmail.hzau.edu.cn (X.L.); 2 Southwest Research Centre for Engineering Technology of Landscape Architecture (State Forestry and Grassland Administration), Southwest Forestry University, Kunming 650224, China; lyourhome@webmail.hzau.edu.cn

**Keywords:** wintersweet (*Chimonanthus praecox* L.), molecular cloning, functional analysis, bHLH transcription factors, terpene production, volatile production

## Abstract

Wintersweet (*Chimonanthus praecox* L.) is an ornamental and economically significant shrub known for its unique flowering characteristics, especially the emission of abundant floral volatile organic compounds. Thus, an understanding of the molecular mechanism of the production of these compounds is necessary to create new breeds with high volatile production. In this study, two *bHLH* transcription factors (*CpMYC2* and *CpbHLH13*) of Wintersweet H29 were functionally characterized to illustrate their possible role in the production of volatile compounds. The qRT-PCR results showed that the expression of *CpMYC2* and *CpbHLH13* increased from the flower budding to full bloom stage, indicating that these two genes may play an essential role in blooming and aroma production in wintersweet. Gas chromatography-mass spectroscopy (GC-MS) analysis revealed that the overexpression of *CpMYC2* in arabidopsis (*Arabidopsis thaliana*) *AtMYC2-2* mutant (Salk_083483) and tobacco (*Nicotiana tabaccum*) genotype Petit Havana SR1 significantly increased floral volatile monoterpene, especially linalool, while the overexpression of *CpbHLH13* in *Arabidopsis thaliana* ecotype Columbia-0 (Col-0) and tobacco genotype SR1 increased floral sesquiterpene β-caryophyllene production in both types of transgenic plants respectively. High expression of terpene synthase (TPS) genes in transgenic *A. thaliana* along with high expression of *CpMYC2* and *CpbHLH13* in transgenic plants was also observed. The application of a combination of methyl jasmonic acid (MeJA) and gibberellic acid (GA3) showed an increment in linalool production in *CpMYC2*-overexpressing arabidopsis plants, and the high transcript level of TPS genes also suggested the involvement of *CpMYC2* in the jasmonic acid (JA) signaling pathway. These results indicate that both the *CpMYC2 and CpbHLH13* transcription factors of wintersweet are possibly involved in the positive regulation and biosynthesis of monoterpene (linalool) and sesquiterpene (β-caryophyllene) in transgenic plants. This study also indicates the potential application of wintersweet as a valuable genomic material for the genetic modification of floral scent in other flowering plants that produce less volatile compounds.

## 1. Introduction

Plants produce a flower aroma that plays a significant role in their economic value and aesthetic properties [1]. Each plant has its own unique floral aroma, consisting of volatile organic compounds, which are mainly derivatives of fatty acids, terpenoids, and phenylpropanoids or benzoids. Thus, an understanding of the mechanism associated with the formation of these floral aromas is necessary to create new breeds, mainly in plants that produce less volatile compounds [2,3]. Two independent and separate pathways, methylerythritol phosphate (MEP) and mevalonic acid (MVA), are responsible for the synthesis of terpenoids [3]. The MVA pathway is involved in the biosynthesis of sesquiterpenes, which accounts for 28% of all floral terpenoids. In contrast, the MEP pathway is mainly involved in the biosynthesis of monoterpene and diterpene, producing approximately 53% and 1% of the total floral terpenoids, respectively [4]. To date, many flower-specific terpene syntheses have been isolated and characterized, such as linalool in *Arabidopsis thaliana*, *Osmanthus fragrans*, *Antirrhinum majus*, *Clarkia breweri*, and *Hedychium coronarium* [4,5,6,7]; myrcene in *Alstroemeria peruviana* and *A. majus* [8]; 1,8-cineole in *Nicotiana suaveolens*, *Citrus unshiu*, and *H. coronarium* [9,10]; *E*-(β)-ocimine in *A. majus* and *H. coronarium* [11,12,13]; sesquiterpene α-farnesene in *Actinidia deliciosa* and *H. coronarium* [14,15]; germacrene D in *Rosa hybrid*, *A. deliciosa*, and *Vitus vinifera* [13,16]; nerolidol in *A. chinensis* and *A. majus* [17]; valence in *V. vinifera* [16]; β-ylangene, β-copaene, β-cubenen, and α-bergamotene in *Cnangaodorata* var. *fruticose* [18], and β-caryophyllene in *A. thaliana*, *Ocimum kilimandscharicum*, and *Daucus carota* [19,20,21]. However, the regulatory mechanisms and the biosynthesis of the floral aroma, especially in woody ornamental plants, are mostly undiscovered.

Jasmonates (JAs), biologically active intermediates involved in the production pathways of jasmonic acid, are associated with a series of biological processes [22] including the ripening of fruits, the production of viable pollen, the growth of roots, tendril coiling, plant responses against the abiotic stress, wounds, and defense against insect pests [23,24,25,26,27,28,29]. JAs are also released as volatile organic compounds (VOCs) and help as a communicator between plants in response to mutual dangers [30]. JAs induce the expression of terpene synthase genes, which results in the release of more terpenoides [31]. The upregulation of terpene synthase (TPS) genes is also related to the development of flowers in plants [32]. In this content, methyl jasmonic acid (MeJA) treatment increases the production of monoterpene (E)-β-ocimine and linalool in tobacco [33]. JAs treatment upregulates the transcription of TPS03 (At4g16740), encoding (E)-β-ocimine synthase in arabidopsis [34]. The MYC family members are basic transcription factors of helix-loop-helix (bHLH) that have numerous functions and are very important in the regulation mechanisms of plants. One of the members of this family, MYC2, has been found to show a significant role in various plant developmental mechanisms including cold stress, insect pest attack, salt stress, salinity, drought, response to light conditions, anthocyanin production, terpene synthesis, and different signaling pathways such as jasmonic acid (JA), gibberellins (GAs), abscisic aid (ABA), auxin (IAA), and salicylic acid (SA) [2,20,21,26,27,35]. MYC2 has also been reported as one of the major players in the crosstalk between JA and GA signaling. It also plays a role as a signal integrator of the JA and GA pathways and was shown to be a positive important regulator of the induction of JA and GA and the expression of terpene synthase genes in *Arabidopsis* [20].

Wintersweet (*Chimonanthus praecox* L.), belonging to the family Calycanthaceae, is a well-known ornamental and economically significant woody plant, commonly known as “Làméi” in China [36,37]. Due to its unique flowering time and strong, pleasant fragrance, it is considered one of the most popular and essential ornamental flowering plants in China [36,38]. Its flowers and natural essential oils have been widely used in traditional Chinese medicine, cosmetics, perfumes, and aromatherapy [39,40]. These traits, along with other characteristics of wintersweet flowers, including flower development, biosynthesis, and aromatic emission, aging, and resistance against biological and abiological stress, make it an important commercial plant [41]. Wintersweet blooms mainly in winter, with a strong fragrance. Therefore, the molecular mechanism of flower development is different from that of plants or species that bloom in spring [39,40,41,42]. Although some attempts have been made to illustrate the molecular processes associated with important floral properties such as senescence, color, and aroma have been extensively investigated in several flowering plants such as *A. majus*, *Rosa gallica, O. fragrans* Lour., and carnation [43], genomic information on wintersweet plants is still limited [44]. Thus, the identification and characterization of functional genes through transcriptome and genome sequencing is vital to uncover the molecular mechanism behind flower fragrance production in wintersweet. Here, we studied two *bHLH* transcription factors (*CpMYC2* and *CpbHLH13*), selected from a transcriptomic library developed from the fully open flower developmental stage of the Wintersweet H29 (Huazhong 29) genotype. The main objectives of this study were molecular cloning and investigating the function of *CpMYC2* and *CpbHLH13.* The effect of these *bHLH* genes on the production of plant floral volatiles was also evaluated.

## 2. Results

### 2.1. Characterization of CpMYC2 and bHLH13 

**Figure 1:**
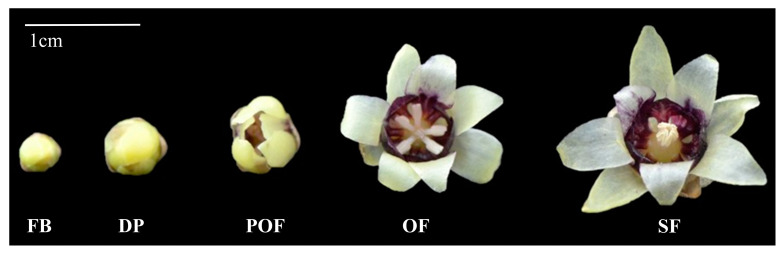
Developing stages of the flower of the Wintersweet (*Chimonanthus praecox*) H29 (Huazhong 29) genotype. Abbreviations: FB, flower bud; DP, display petal; POF, partially-open flower; OF, open flower stage; SF, senescing flower. Scale bar = 1 cm.

**Figure 2:**
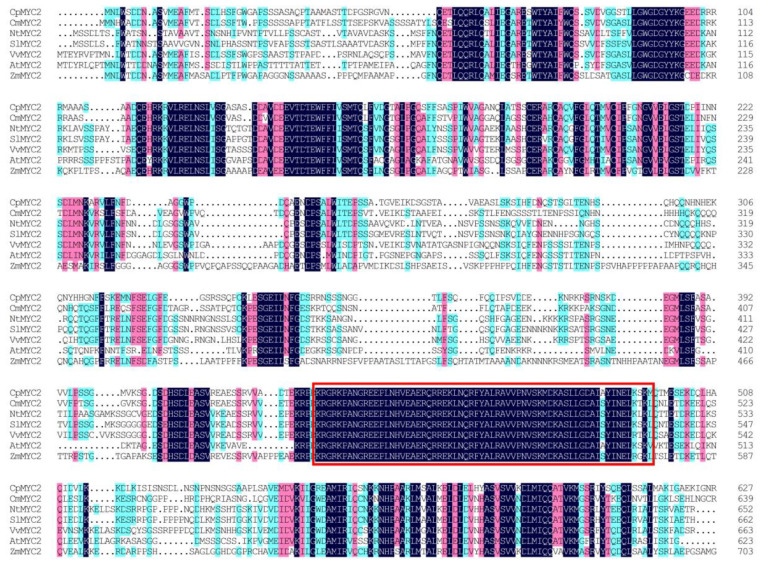
Multiple sequence alignment of the wintersweet *CpMYC2* protein sequence with its homologous proteins from other plant species: *Cinnamomum micranthum* CmMYC2 (RWR86802.1), *Nicotiana tabacum* NtMYC2 (XP_016500373.1), *Solanum lycopersicum* SlMYC2 (NP_001311412.1), *Vitis vinifera* VvMYC2 (XP_002280253.2), *Arabidopsis thaliana* AtMYC2 (At1g32640), and *Zea mays* ZmMYC2 (PWZ55921.1). The red box indicates the basic helix-loop-helix DNA-binding domain common in plants.

**Figure 3:**
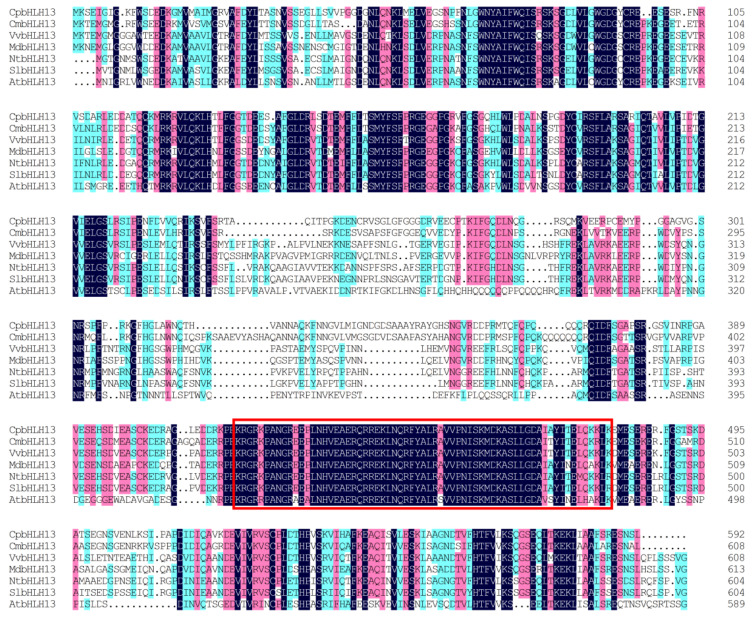
Multiple sequence alignment of the Wintersweet *bHLH13* protein sequence with its homologous proteins from other plant species: *Cinnamomum micranthum* CmbHLH13 (RWR96436.1), *Vitis vinifera* VvbHLH13 (RVW95465.1), *Malus domestica* MdbHLH13 (XP_028955372.1), *Nicotiana tabacum* NtbHLH13 (XP_016459177.1), *Solanum lycopersicum* SlbHLH13 (XP_004229991.1), and *Arabidopsis thaliana* AtbHLH13 (AT1G01260). The red box indicates the basic helix-loop-helix DNA-binding domain common in plants.

Using the Illumina RNA-Seq technique, a transcriptomic library (NCBI Sequence Read Archive (SRA) accession number PRJNA492170) was constructed from the full open flower stage of the Wintersweet H29 cultivar (Figure 1), and out of 16 identified members of the *bHLH* family, the desired sequence of two transcription factors *CpMYC2* (2858 bp) and *bHLH13* (2425 bp) was selected for further study [45]. Based on the NCBI ORF finder, the open reading frame of 1884 bp and 1779 bp nucleotide, encoding 628 and 593 amino acids, were observed in *CpMYC2* and *CpbHLH13*, respectively. The theoretical isoelectric point (pI) and the molecular weight (Mw) of the *CpMYC2* and *CpbHLH13* proteins were Pi/Mw: 5.65/68707.29 and Pi/Mw: 6.43/65502.53, respectively. Multiple sequence alignment of wintersweet *CpMYC2* and *CpbHLH13* proteins with their homologous protein sequences in other species, especially *A. thaliana*, *Nicotiana tabacum*, *V. vinifera*, *Cinnamomum micranthum*, *Solanum lycopersicum*, and *Zea mays* revealed the presence of a basic helix-loop-helix DNA-binding domain (446–493 amino acids for *CpMYC2* and 429–476 amino acids for *CpbHLH13*) with an MYC-N terminal, which is a common feature of MYC family proteins (Figure 2 and Figure 3). The three-dimensional protein structure of *CpMYC2* and *CpbHLH13* is shown in Supplementary Figures S1 and S2. Phylogenetic analysis demonstrated that *CpMYC2* was closely related to the transcription factor Arabidopsis MYC2 *(AT1G32640)*, whereas *CpbHLH13* was most homologous to *AtbHLH13 (AT1G01260)*, also known as JASMONATE-ASSOCIATED MYC2-like2 (JAM2) (Supplementary Figures S3 and S4). Further phylogenetic analysis with different species revealed that *CpMYC2* had a close relationship with the predicted protein of *Cinnamomum micranthum* (*CmMYC2*) with 65.13% identity and *A. thaliana* (*AtMYC2*) with 54%, while *CpbHLH13* was mostly homologous to *C. micranthum* (*CmbHLH13*) with 69.77% identity followed by *Nelumbo nucifera* (*NnMYC2*) with 62.46% identity and *A. thaliana AtbHLH13* with 42% identity, although they were not in the same clade. Phylogenetic analyses revealed that *CpbHLH13* had a close relationship with the *A. thaliana AtbHLH13*/JAM2 (At1g01260) and *AtbHLH17*/JAM1 (At2g46510) transcription factors (See Supplementary Figures S5 and S6). A summary of the homology of wintersweet *CpMYC2* and *CpbHLH13* with identity% and functions of the genes from various plants is described in Table 1 and Table 2.

### 2.2. CpMYC2 and CpbHLH13 Expression in Wintersweet

**Figure 4:**
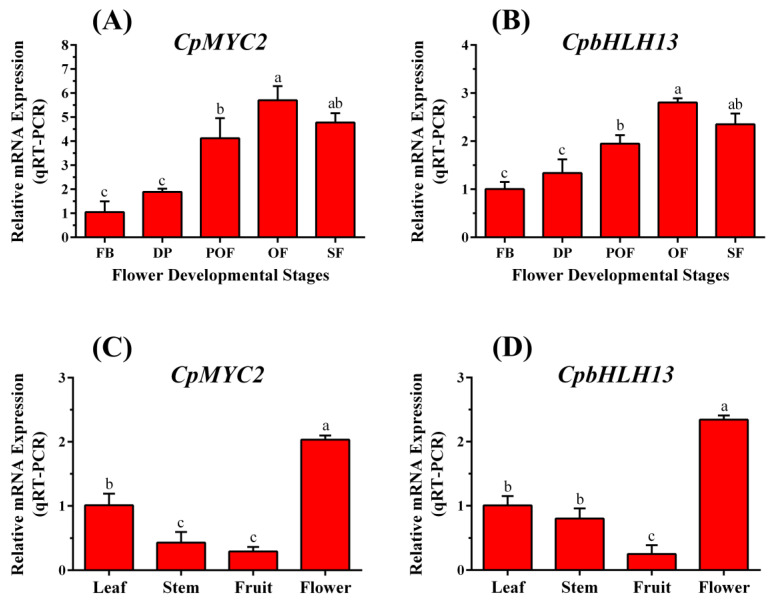
The transcriptomic expression pattern of (**A**) *CpMYC2* and (**B**) *CpbHLH13* during flower developing stages of H29. The stages are FB: flower bud, DP: display petal, POF: partially-open flower, OF: open flower, SF: senescing flower. Tissue-specific analysis of (**C**) *CpMYC2* and (**D**) *CpbHLH13* expression in wintersweet. The different letters on the bars show significance between the treatments by using least significant difference (LSD) at *p* < 0.05.

To determine whether *CpMYC2* and *CpbHLH13* expression was correlated with VOC biosynthesis during wintersweet inflorescence, the expression profile of *CpMYC2* and *CpbHLH13* was analyzed in five developing stages of the flower of H29: flower bud (FB) stage, display petal (DP) stage, partially-open flower (POF) stage, open flower (OF) stage and senescing flower (SF) stage (Figure 1) using qRT-PCR. The results indicated that the expression levels of *CpMYC2* and *CpbHLH13* increased from the budding stage of the flower to the fully-open flower stage and then gradually decreased in the senescing stage (Figure 4a,b). We also measured the expression level of both the genes in different tissues of wintersweet and found that the expression level of these genes was highest in flowers, followed by leaves and stems, and lowest in fruits (Figure 4c,d). This indicates their importance and possible involvement in producing VOCs during blooming, also indicating that these two genes may play an important role in blooming and aroma production.

### 2.3. Expression Analysis of the Terpene Synthase Genes in Wild-Type and Transgenic Plants

**Figure 5:**
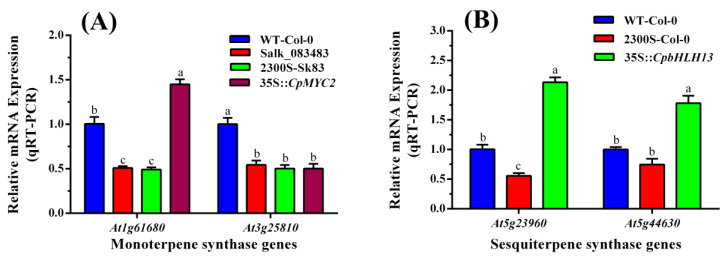
Expression of (**A**) the monoterpene synthase genes *At1g61680* and *At3g25810* in arabidopsis wild type (WT-Col-0), mutant (Salk_083483), and mutant Salk_083483 transformed by the empty vector pCAMBIA2300S (2300S-Sk83) and overexpressed *CpMYC2* gene (35S::*CpMYC2*) plants and (**B**) the sesquiterpene synthase genes *At5g23960* and *At5g44630* in arabidopsis wild type (WT-Col-0), wild type transformed by the empty vector pCAMBIA2300S (2300S-Col-0) and overexpressed *CpbHLH13* gene (35S::*CpbHLH13*) plants measured by qRT-PCR. The different letters on the bars show significance between the treatments by using least significant difference (LSD) at *p* < 0.05.

Real-time PCR (qRT-PCR) was used to measure the expression of terpene synthase genes in wild-type and transgenic plants. The results indicate that the expression of the linalool-producing gene *At1g61680* was about one and a half times higher in the plants transformed by *CpMYC2* than the other plants, while wild-type Col-0 plants, the mutant Salk_083483, and mutant plants transformed by the empty vector pCAMBIA2300S (2300S-Sk83) also showed lower expression of the *At3g25810* gene as compared to 35S::*CpMYC2* plants (Figure 5a). Similarly, the expression of the β-caryophyllene-producing genes *At5g23960* and *At5g44630* was almost threefold higher in the plants transformed by the *CpbHLH13* gene than the wild-type arabidopsis plants (Col-0) and the plants transformed by the vector pCAMBIA2300S (Figure 5b). The expression of the *CpMYC2* and *CpbHLH13* genes in transgenic tobacco and arabidopsis plants was also measured (Supplementary Figure S7) and the plants with almost identical expression were used for further experimentation.

### 2.4. Overexpression of CpMYC2 and CpbHLH13 Induces VOC Emission

**Figure 6:**
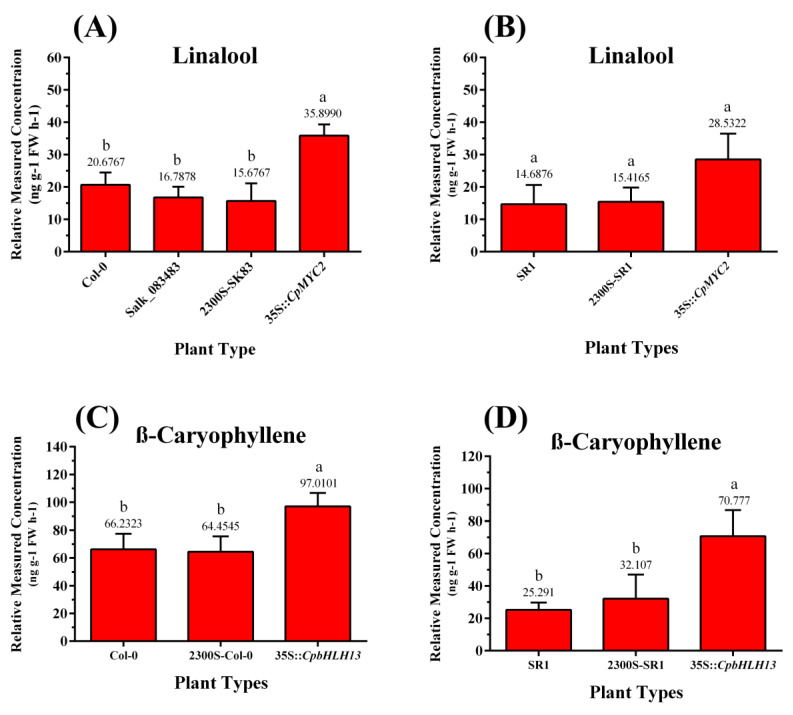
The concentration of emitted linalool (ng g-1 FW h-1) from (**A**) arabidopsis wild type (WT-Col-0), mutant (Salk_083483), mutant Salk_083483 transformed by the empty vector pCAMBIA2300S (2300S-Sk83) and overexpressed *CpMYC2* gene (35S::*CpMYC2*), and (**B**) tobacco wild type SR1, transformed by the empty vector pCAMBIA2300S (2300S-SR1) and overexpressed *CpMYC2* gene (35S::*CpMYC2*) plants. Concentration of emitted β-caryophyllene (ng g-1 FW h-1) from (**C**) arabidopsis wild type (WT-Col-0), wild type transformed by empty vector pCAMBIA2300S (2300S-Col-0) and overexpressed *CpbHLH13* gene (35S::*CpbHLH13*), and (**D**) tobacco wild type SR1, transformed by the empty vector pCAMBIA2300S (2300S-SR1) and overexpressed *CpbHLH13* gene (35S:*:CpbHLH13*) plants. The different letters on the bars show significance between the treatments by using least significant difference (LSD) at *p* < 0.05.

**Figure 7:**
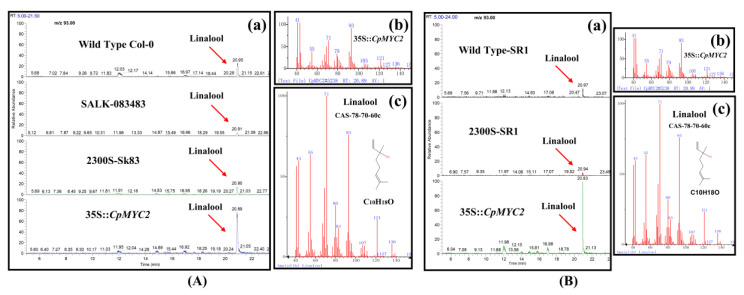
GC-MS analysis of linalool emitted from the flowers of (**A**) arabidopsis wild type (WT-Col-0), mutant Salk-083483, transformed by the empty vector pCAMBIA2300S (2300S-Sk83) and overexpressed *CpMYC2* gene (35S::*CpMYC2*) plants, and (**B**) tobacco wild type SR1, transformed by the empty vector pCAMBIA2300S (2300S-SR1) and overexpressed *CpMYC2* gene (35S::*CpMYC2*) plants. (**a**) GC-MS chromatogram comparison between different plants. (**b**) Mass spectrum of the product. (**c**) Product confirmation in the NIST database according to retention index and mass spectrum.

**Figure 8:**
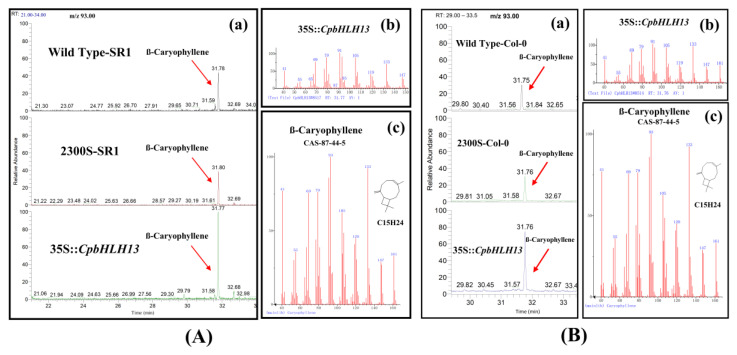
GC-MS analysis of β-caryophyllene emitted from the flowers of (**A**) tobacco wild type SR1, transformed by the empty vector pCAMBIA2300S (2300S-SR1) and overexpressed *CpbHLH13* gene (35S:*:CpbHLH13*) plants; (**B**) arabidopsis wild type (WT-Col-0), transformed by the empty vector pCAMBIA2300S (2300S-SR1) and overexpressed *CpbHLH13* gene (35S:*CpbHLH13*) plants. (**a**) GC-MS chromatogram comparison between different plants. (**b**) Mass spectrum of the product. (**c**) Product confirmation in the NIST database according to retention index and mass spectrum.

The positively selected plants obtained from T3 generation were used to detect the terpenes from the inflorescence of transgenic arabidopsis and tobacco plants using GC-MS. We analyzed the emission of terpenes in arabidopsis and tobacco transgenic plants overexpressed with the *CpMYC2* and *CpbHLH13* gene. The results indicate that linalool was the most abundant monoterpene (Figure 6a,b) and its emission was more than 1.5 and 2 times higher in transgenic arabidopsis and tobacco plants transformed by the *CpMYC2* gene (35S::*CpMYC2*), respectively, compared to the wild type (Col-0 and SR1) and transgenic plants transformed by the vector pCAMBIA2300S (Figure 7a,b). Similarly, it was found that β-caryophyllene was the most abundant sesquiterpene produced in arabidopsis and tobacco plants transformed by the *CpbHLH13* gene (Figure 6c,d). GC-MS analysis of linalool and β-caryophyllene emitted from the flowers of transformed *A. thaliana* and tobacco plants is shown in Figure 7 and Figure 8. The results also indicate that the emission of β-caryophyllene was about twofold higher in the plants transformed by the *CpbHLH13* gene than the transgenic plants transformed by the vector pCAMBIA2300S and wild-type tobacco SR1 (Figure 8a,b).

### 2.5. Application of MeJA and GA3 on Overexpressed CpMYC2 Plants

**Figure 9:**
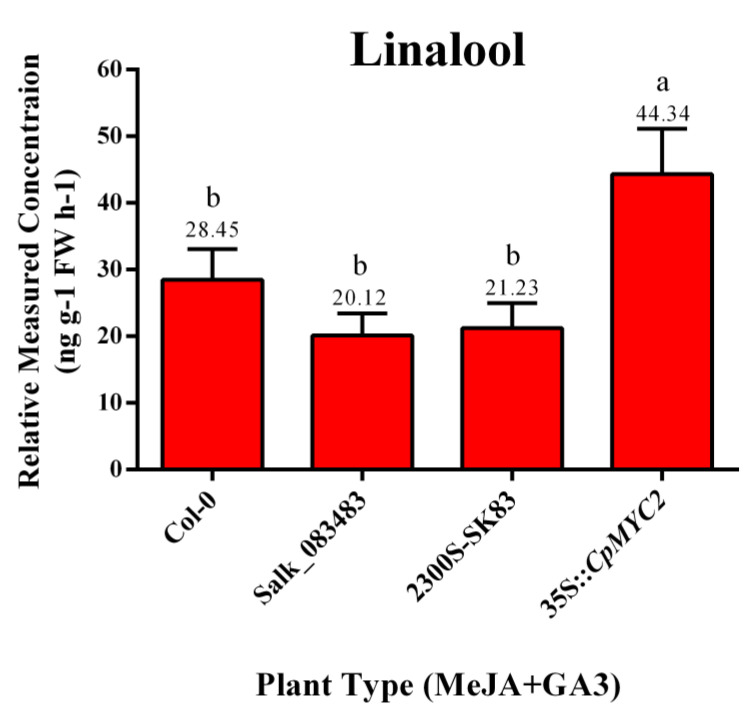
The concentration of emitted linalool (ng g-1 FW h-1) after the application of methyl jasmonic acid (MeJA) and gibberellic acid (GA3) from arabidopsis wild type (WT-Col-0), mutant (Salk_083483), mutant Salk_083483 transformed by the empty vector pCAMBIA2300S (2300S-Sk83), and *CpMYC2* gene (35S::*CpMYC2*) plants. The different letters on the bars show significance between the treatments by using least significant difference (LSD) at *p* < 0.05.

**Figure 10:**
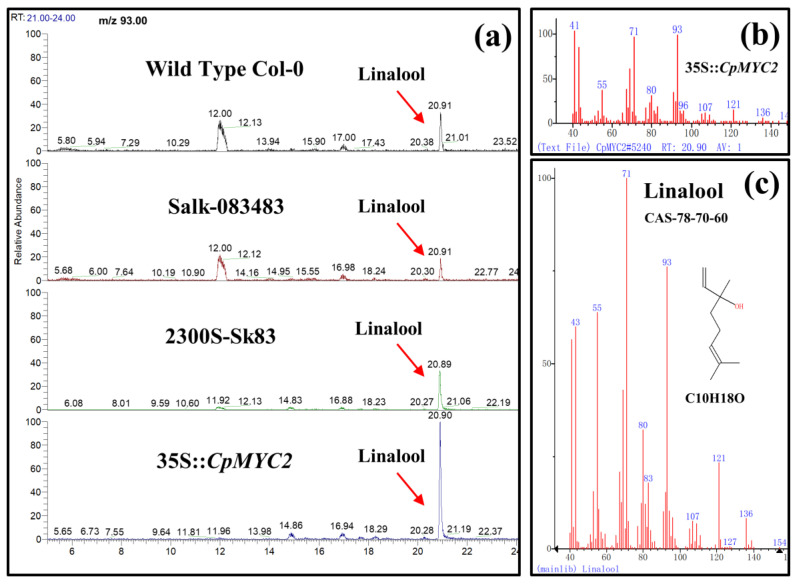
GC-MS analysis of linalool emitted after the application of MeJA and GA3 from the flowers of arabidopsis wild type (WT-Col-0), mutant (Salk_083483), mutant Salk_083483 transformed by the empty vector pCAMBIA2300S (2300S-Sk83), and *CpMYC2* gene (35S::*CpMYC2*) plants. (**a**) GC-MS chromatogram comparison between different plants. (**b**) Mass spectrum of the product. (**c**) Product confirmation in the NIST database according to retention index and mass spectrum.

**Figure 11:**
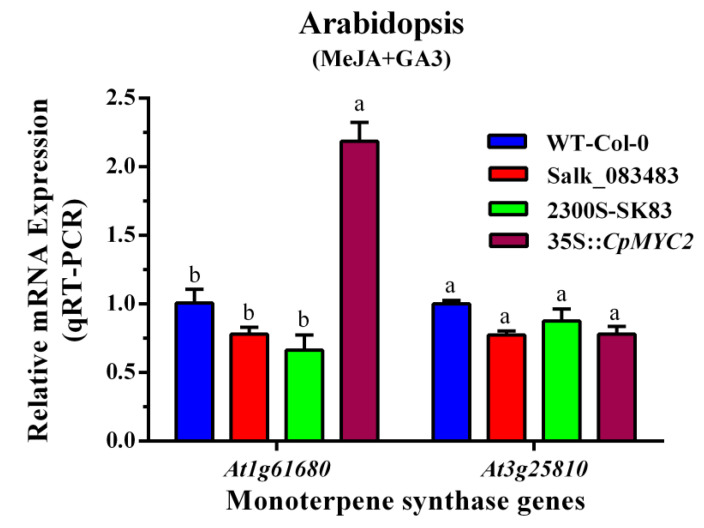
Expression of the monoterpene synthase genes *At1g61680* and *At3g25810* in arabidopsis plants by qRT-PCR after the application of MeJA and GA3 in arabidopsis wild type (WT-Col-0), mutant (Salk_083483), mutant Salk_083483 transformed by the empty vector pCAMBIA2300S (2300S-Sk83), and *CpMYC2* gene (35S::*CpMYC2*) plants measured by qRT-PCR. The different letters on the bars show significance between the treatments by using least significant difference (LSD) at *p* < 0.05.

Following MeJA and gibberellic acid (GA3) treatment, terpenoids were detected in the inflorescence of transgenic arabidopsis plants using GC-MS. The results again indicate that the emission of linalool was more than 1.5 times higher in the plants transformed by the *CpMYC2* gene (35S::*CpMYC2*), while the mutant plants Salk_083483, mutant plants transformed by the vector pCAMBIA2300S (2300S-Sk83), and the Col-0 wild-type plants showed a lower amount of linalool emission compared to the wild-type (35S::*CpMYC2*) plants (Figure 9 and Figure 10). Further analysis using real-time PCR (qRT-PCR) indicated that the expression of the linalool-producing gene *At1g61680* was about twofold higher in the plants transformed by *CpMYC2* (35S::*CpMYC2*) than the other plants, while the wild-type Col-0, mutant plants Salk_083483, and mutant plants transformed by the vector pCAMBIA2300S (2300S-Sk83) showed a lower expression of the *At3g25810* gene as compared to 35S::*CpMYC2* plants (Figure 11).

### 2.6. Overexpression of CpbHLH13 Reduces Flower Pigmentation in Tobacco Plants 

**Figure 12:**
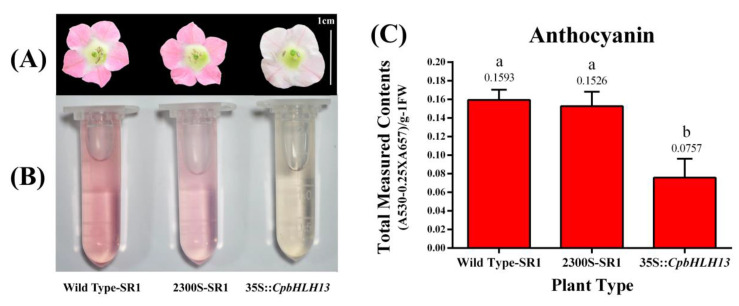
Anthocyanin accumulation from the flowers of (**A**) overexpressed *CpbHLH13* tobacco plants (35S:*CpbHLH13*), wild type tobacco SR1 (Wild Type-SR1), and tobacco plants transformed by the empty vector pCAMBIA2300S (2300S-SR1). (**B**) Anthocyanin detection from the described plants. (**C**) Quantitative anthocyanin concentration in tobacco plants. Scale bar = 1 cm. The different letters on the bars show significance between the treatments by using least significant difference (LSD) at *p* < 0.05.

A few of the flowering transformed lines of *CpbHLH13* also showed phenotypic changes in petal pigmentation. These transgenic plants showed a reduction in the pigmentation of flowers (Figure 12a). Anthocyanin contents from the inflorescence of these tobacco plants were measured by a spectrophotometer which showed a clear reduction in the anthocyanin concentration in 35S::*CpbHLH13* plants compared with those of the wild type and those transformed by the empty vector pCAMBIA2300S (2300S-SR1) (Figure 12b,c). By measuring the anthocyanin contents in *CpbHLH13* gene-transferred transgenic tobacco (35S::*CpbHLH13*) plants, we confirmed the reduction of anthocyanin contents in their inflorescence.

## 3. Discussion

Plants can store and release a wide range of volatile compounds through their specialized organs and tissues, which play a significant role in atmospheric composition [46]. These volatile compounds are also important for the formation of the characteristic floral scent in different plants which improves the aesthetic properties as well as the economic value of the plant [15]. However, the regulatory mechanisms behind the formation of the floral aroma in ornamental plants are poorly characterized. To date, no bHLH transcription factor has been identified and verified by its function in the ornamental and economically significant shrub “wintersweet”. A few attempts have been made to illustrate the transcriptomic analysis of these genes, but the functional characterization remains underestimated. This might be due to the lack of genetic resources as well as the difficulties in the transformation of the genes in wintersweet [9,47]. The present study was designed to decipher the molecular mechanism behind flower fragrance production in wintersweet. The motives behind the study were to identify and find out the role and function of the bHLH transcription factors *CpMYC2* and *CpbHLH13* in wintersweet.

Phylogenetic analysis demonstrates that *CpMYC2* is closely related to Arabidopsis MYC2 (*AT1G32640*), whereas *CpbHLH13* is most homologous to *AtbHLH13* (*AT1G01260*), also known as JASMONATE-ASSOCIATED MYC2-like2 (JAM2). MYC2 was previously reported to promote and activate the sesquiterpene synthase genes TPS21 and TPS11 through interacting with the DELLA proteins (MYC2 interacts with DELLA proteins and regulates the expression of the sesquiterpene synthase gene). It has also been reported that MYC2 is directly involved in the induction of JA and GA in sesquiterpene biosynthesis [48] and induces monoterpene synthase in arabidopsis, which suggests that MYC2 endorses the biosynthesis of a wider range of terpenes [49], whereas JAM2 (*AtbHLH13*) is involved in the negative regulation of jasmonate signaling in plants [50]. They have also been reported as novel factors regulating various metabolic pathways in arabidopsis in JA signaling [51]. It has been reported that MYC2 is differentially regulated and induced during the flower opening of wintersweet, especially at the fully-open flower stage [37]. In the present study, high expression of both the genes was found at the fully-open flower stage compared to the other flower developmental stages along with the highest expression in flowers among the other developing tissues of the plant (leaves, stems, and fruits). This indicates the possible role of these genes in volatile production during flowering. MYC2 has been identified to enhance the production of volatile compounds, especially monoterpenes and sesquiterpenes, in plants [20,52]. The expression of the linalool- and β-caryophyllene-producing genes increases in plants transformed by *CpMYC2* and *CpbHLH13*, respectively. This illustrates the role of the transcription factors *CpMYC2* and *CpBHLH13* in the production of monoterpenes and sesquiterpenes in wintersweet plants. 

Our findings also indicate that the overexpression of *CpMYC2* and *CpbHLH13* in model plants shows an increase in the production of monoterpene, i.e., linalool as well as the sesquiterpene (E)-β-caryophyllene, respectively. It is possible that the significant increment in compounds could be provoked because it is a MYC2 transcription factor and part of the bHLH family, which is involved in the regulation of many regulatory functions of the plant and also involved in plant growth and the mechanism of flower development in plants [22,30,53,54]. The results also show that *CpMYC2* possibly increases the production of linalool by inducing the expression of the linalool- and limonene-producing genes *At1g61680* and *At3g25810* in arabidopsis. Linalool itself is one of the main produced floral volatiles, and the sequestration of linalool derivatives and the derivatives of the isoteric monoterpene alcohol geraniol were experimentally proved to be present in the floral organs of several plant species. 

The present study also indicates the possible involvement of wintersweet *CpbHLH* genes in the signaling pathways of JA and GA, leading to the production of volatile floral terpenes. A similar phenomenon was reported in other plant transcription factors such as *AtPAP1* in *Rosa hybrida*, WRKY in cotton (*Gossypium hirsutum*), ORCA3 in *Catharanthus roseus*, EF1 and ERF2 in *Atemisia annua*, and *AtMYC2* in arabidopsis [20,55,56,57,58]. These transcription factors regulate the TPS genes by interacting with the DELLA proteins. The DELLA proteins act as a repressor of the TPS gene and negatively regulate the JA and GA signaling pathways by interacting with MYC2. An increase in the concentration of JA and GA decreases the level of JAZ and DELLA proteins, thus releasing the MYC2 and inducing the TPS genes, which results in the production of more volatile compounds [20,59,60]. A combined application of JA and GA3 results in an increase in the expression of the TPS gene and volatile compound production in the transformed plants. Our findings are consistent with the previously reported experimental results where the application of MeJA induces monoterpene production and emits (E)-β-ocimine, β-myrcene, and a small increase in linalool in transgenic tobacco plants [60]. The plants transferred with the *CpbHLH13* gene were found to increase sesquiterpene β-caryophyllene production in the arabidopsis and tobacco plants, and the expression of the genes *At5g23960* and *At5g44630*, which are involved in the production of almost all the sesquiterpenes in the arabidopsis, was also higher than in non-transformed plants. It was previously reported that the arabidopsis gene *At5g23960* is associated with the synthesis of humulene and β-caryophyllene, which together produce 43% of the terpene volatiles from the flower [49].

In addition to increase sesquiterpene (β-caryophyllene) production in the arabidopsis and tobacco plants, we also found a few transgenic tobacco plants (35S::*CpbHLH13*) showing reduced flower color compared with the wild type and those transformed by the empty vector pCAMBIA2300S (2300S-SR1). The results were also verified by measuring anthocyanin contents from the flower samples using a spectrophotometer. This significant decrease in anthocyanin production could be explained by the previously described function of the bHLH13 (JAM2) and bHLH17 (JAM1) transcription factors that act as the transcriptional repressors which negatively regulate JA responses resulting in anthocyanin reduction in transgenic plants [50,51,61,62,63]. Furthermore, it has also been reported that bHLH17 is a bHLH subgroup III transcription factor which acts as a transcriptional repressor after binding to the promoters of the target genes in the anthocyanin-regulating pathway [64], which antagonizes the activation function of MYC2 and TT8/MYB75 to negatively regulate JA responses including flowering and anthocyanin accumulation [61].

It has also been elucidated by a functional study that JAZ interacts with transcriptional activators such as MYC2 and MYB75, along with the transcriptional repressors bHLH13 and bHLH17, to reduce its transcriptional functions [61]. The balance between activators and repressors leads to an adequate output of JA responsive genes, which results in a suitable level of JA responses such as plant defense and anthocyanin accumulation.

In this study, we characterized the functional aspects of two wintersweet bHLH transcription factors, *CpMYC2* and *CpbHLH13*, for the production of volatile organic compounds. The results show that *CpMYC2* could increase the production of monoterpenes such as linalool, and *CpbHLH13* could increase the production of sesquiterpenes such as β-caryophyllene in transgenic plants by enhancing the activity of terpene-producing genes. The application of the MeJA with GA3 could promote the production of linalool and linalool-producing genes in arabidopsis. Taken together, the findings from this study reveal that bHLH transcription factors (*CpMYC2* and *CpbHLH13*) play an essential role in the production of aroma in wintersweet. This study also indicates the future potential application of wintersweet as a valuable genomic material for the genetic modification of floral scent in other flowering plants that produce small amounts of volatile organic compounds.

## 4. Materials and Methods

### 4.1. Selection and Analysis of Candidate Genes

The sequences of two bHLH candidate genes predicted as MYC2 and bHLH13 were obtained from a transcriptomic library (NCBI Sequence Read Archive (SRA) accession number PRJNA492170) constructed from the fully open flower of the Wintersweet (*C. praecox* L.) H29 (Huazhong 29) genotype situated in the garden of Huazhong Agricultural University, Wuhan, China [45]. Moreover, these genes were verified as full-length coding sequences (CDS) from the genomic data of the H29 cultivar (unpublished data) with no introns inside the sequences using BioEdit 7.0 [65] and UltraEdit software [66]. The nucleotide sequences of both candidate genes are shown in supplementary data (Sequences S1 and S2). Multiple sequence alignment of selected bHLH transcription factors of Wintersweet and other plants was performed using the CLUSTALW program with default parameters [67]. The phylogenetic tree was constructed using the neighbor-joining method with 1000 bootstrap replicates using MEGA X software [68]. These candidate genes were confirmed as transcription factors and named *CpMYC2* and *CpbHLH13* as per their homology with the typical MYC2 and bHLH13 transcription factor proteins of other plants. The homology of the gene sequence with arabidopsis was also confirmed by the arabidopsis information resource (TAIR) database (https://www.arabidopsis.org/Blast/index.jsp). The conserved domain of the *CpMYC2* and *CpbHLH13* gene was observed (https://www.ncbi.nlm.nih.gov/guide/domains-structures) using Pfam (http://pfam.xfam.org/search/sequence), and protein structure was predicted using Swissmodel (https://swissmodel.expasy.org/interactive). The Compute pI/Mw calculation tool on the ExPASy server (https://web.expasy.org/compute_pi) was used to predict the theoretical isoelectric point (pI) and the molecular weight (Mw).

### 4.2. Plant Materials

Flowers of the Wintersweet H29 plant were collected from the garden of Huazhong Agriculture University in Wuhan, China. Plants of the *Arabidopsis thaliana* ecotype Columbia (Col-0), the T-DNA inserted homozygous myc2-2 (Salk_083483) mutant [69] obtained from the SALK institute and ABRC (Arabidopsis Biological Resource Center, Columbus, OH, USA), and the Nottingham Arabidopsis Stock Centre and tobacco (*Nicotiana tabacum*) cv Petit Havana SR1 [70] were used as the planting and gene-transforming material. The homozygosity of the myc2-2 (Salk_083483) mutant arabidopsis plants was detected by the specific primers listed in Table 3. Plants were grown in half-strength Murashige and Skoog (MS) media under a 16-h photoperiod in the plant growth chamber.

### 4.3. Total RNA Extraction, cDNA Synthesis, and Quantitative Real-Time PCR (qRT-PCR) Analysis

Total RNA was extracted from 0.20g frozen flowers using TRIzol reagent (CoWin Biotech Co., Ltd., Beijing, China) as per the manufacturer’s instructions. DNA contamination was removed by treating the total RNA with the DNase I enzyme. cDNA was synthesized using a one-step gDNA removal and cDNA synthesis supermix (Transgene, Wuhan, China) according to the manufacturer’s instructions. The synthetic first-strand cDNAs were diluted 10-fold for gene expression analysis. The gene expression of *CpMYC2* and *bHLH13* was studied by qRT-PCR in 5 developing stages of the flower of H29, i.e., FB (flower bud), DP (display petal), POF (partially-open flower), OF (open flower stage), and SF (senescing flower) (Figure 1) on an Applied Biosystems 7500 Fast Real-Time PCR platform with the SYBR Premix Ex Taq^TM^ II mix (Takara Biotechnology Co., Ltd., Dalian, China), according to the manufacturer’s instructions, and the results were analyzed using the Applied Biosystems 7500 software (Applied Biosystems Life Technologies). Three biological replicates were tested, and reactions carried out in triplicate. Relative transcript levels were calculated by the 2^−∆∆Ct^ method [71] using the previously reported most stable genes *RPL8* and *Tubulin* (*CpTublin*) for wintersweet [72,73], *NADH* dehydrogenase *(NtNADH*) and chaperonin *CPN60-2* (*NtCPN60-2*) for tobacco [74], and clathrin adaptor complex subunit (CACS) (*AtCACS*) and β-tubulin2 (*AtTubulin*) [20,75] for arabidopsis as the endogenous control genes for data normalization according to the recommendations [76] and described method [77]. The primers 35S F and 35S R were used for the expression of empty gene transformation. The transcript level of monoterpene-producing *At1g61680* and *At3g25810* along with the sesquiterpene-producing *At5g23960* and *At5g44630* genes in arabidopsis was also measured. Primer Premier 5 software was used to design the primers used in the experiments. The primers used for qRT-PCR analysis are listed in Table 4.

### 4.4. Isolation, Cloning, and Sequencing of Candidate Genes

The full-length open reading frame of *CpMYC2* and *CpbHLH13* was amplified by PCR from the cDNA of wintersweet flowers by using a specific pair of primers containing Kpn1 and Xba1 restriction sites (Table 5). The PCR conditions were 5 min, 95 °C; 30 s, 95 °C; 30 s, 60 °C, 30 cycles; 30 s, 72 °C; 5 min, 72 °C. The PCR product was purified using a Tiangen Midi Purification Kit (Tiangen, Wuhan, China) and cloned into a pEASY-T1 cloning vector (Transgene Wuhan, China), according to the manufacturer’s instructions, and three positive clones were selected and sequenced.

### 4.5. Expression Vector Construction and Transformation

The expression vector was constructed using full-length *CpMYC2* and *CpbHLH13* gene sequences on a modified pCAMBIA 2300S vector by double digestion using Kpn1 and Xba1 digestion enzymes under a CaMV 35S promoter (Supplementary Figure S8a,b); 35S::*CpbHLH13* was introduced into *Agrobacterium tumefaciens* strain GV3101 by electroporation. The positively cloned agrobacteria were selected and transformed into tobacco by using the leaf disk method [78]. The empty vector pCAMBIA 2300S was also introduced into tobacco plants as a control. The shoots of emerged explants were transferred into rooting media as described previously [78]. These explants were incubated in a growth chamber at 25 °C under a 16 h/8 h photoperiod. Homozygous arabidopsis Salk_083483 mutant plants were transformed by *CpMYC2*, while *CpbHLH13* was transferred into the arabidopsis Col-0 cultivar via *Agrobacterium tumefaciens*-mediated transformation [79]. The pots containing the wild-type and mutant arabidopsis plants were carefully inverted and immersed in the infiltration medium containing agrobacterium while stirring for 1 min. The seedlings were placed back on the shelf and covered by plastic bags. Three days after infiltration, the plastic bags were removed, and the seedlings were supported with sticks and allowed to grow till the first generation of seeds (T1). The seeds from the T1 generation of arabidopsis plants and the T0 generation from tobacco were grown in ½ MS media plates with 50 µM-mL-1 kanamycin for screening the positive seedlings. The surviving plants were transferred to soil pots, and their positivity was also confirmed by PCR with gene-specific primers using plant cDNA as a template (Table 5). These plants were self-crossed, and at least three plants from tobacco and 3 groups of 6 plants from arabidopsis of the T3 generation with the highest gene expression were used for further experiments.

### 4.6. Volatile Organic Compounds Analysis

The released floral volatiles from tobacco plants were collected by solid-phase microextraction (SPME) [43]. In triplicate, 2 g fresh flowers were placed into a 20 mL capped SPME vial and incubated at 25 ± 2 °C for 30 min. SPME fiber (50/30 μm DVB/CAR/PDMS on a 2 cm Stable Flex fiber, Supelco Inc., Bellefonte, PA, USA) was then exposed to the headspace of the capped vial for 30 min. The fiber was injected manually and desorbed in the injection port of the gas chromatograph (GC) with helium as the carrier gas. The fiber was desorbed for 5 min at 250 °C in splitless mode. Before each set of samples was assayed, the fiber was conditioned for 1 h at 250 °C in the injection port of the GC-MS and a fiber blank recorded. To measure the release of volatiles by arabidopsis plants, the closed-loop stripping method [80] was used in which the intact rosette plants with their root balls were wrapped with aluminum foil and placed in 3-L bell jars under controlled growth conditions as described previously [49]. VOCs were collected in daytime for 8 h using 1.5 mg activated charcoal and eluted with 40 μL of CH_2_Cl_2_. The eluted samples of 1μL were injected in splitless mode into a TRACE GC Ultra GC coupled to a DSQ II mass spectrometer (Thermo Fisher Scientific, Waltham, MA, USA) equipped with an HP-5 MS fused-silica column (30 m × 0.25 mm × 0.25 μm) (Agilent Technologies, http://www.agilent.com). The GC-MS was performed according to the standard methods [43]. The relative quantification of the target compounds for emission was determined using standard curves which were generated by three repeats: y = 7969151.04x-4406492.42 and R^2^ = 0.97 for linalool and y = 50591253.45x-45934339.38 and R^2^ = 0.99 for β-caryophyllene (Supplementary Figure S9a,b).

### 4.7. Application of MeJA and GA3 to Arabidopsis Plants

We applied MeJA (50 µM) together with GA3 (50 µM) on the arabidopsis wild type (Col-0), mutant (Salk_083483), mutant plants transformed by the empty vector pCAMBIA2300S (2300S-SK83), and mutant plants transformed by the *CpMYC2* gene (35S::*CpMYC2*). The pots, in which 5-week-old plants were growing (T3 generation), were soaked with MeJA (50 mM) and GA3 (50 mM) solutions, respectively, for an indicated time, accompanied with the spraying of aerial parts at intervals of 1 h. dH_2_O_2_ was used as a mock treatment. Inflorescences were harvested for RNA extraction. The hormone treatments for gene analysis were performed in the daytime for 4 h (10:00 to 14:00), and those for volatile analysis were 6 h (9:00 to 15:00).

### 4.8. Anthocyanin Contents Measurements

Total anthocyanin contents were measured by the method described [81]. Flower material (500 mg) from each treatment was crushed in a mortar with liquid nitrogen to obtain a fine powder. The anthocyanins were extracted by transferring the fine powder to an extraction solution (5 mL) containing a methanol/HCl mixture (99:1 *v*/*v*) (Sigma, St. Louis, MO, USA). The mixture was incubated at 4 °C for 24 h and then centrifuged at 13,000 rpm for 20 min at 4 °C. The supernatant was transferred to a fresh tube and the total anthocyanin was determined by measuring the OD at A530 (λ_max_ for anthocyanin) and A657 (peak of absorption for chlorophyll) by using a spectrophotometer (Shimadzu, Kyoto, Japan). The quantification of anthocyanins was performed using the formula Q_anthocyanins_ = (A530 − 0.25 × A657) × M−1, where Q_anthocyanins_ is the amount of anthocyanins, and M is the weight (g) of the plant material used for extraction, as described earlier [82].

### 4.9. Statistical Analysis

All the statistical analysis was conducted using the Statistix 8.1 (FL, USA) software. One-way analysis of variation (ANOVA) followed by Tukey’s HSD multiple comparisons were used to compare the relative expression of transcripts. Three technical and biological repeats were selected separately for every experiment. *p*-value < 0.05 was considered as significant.

## Figures and Tables

**Table 1:** Summary of the homology of wintersweet *CpMYC2* with genes from other plants.

Plant Name	Gene	Identity %	Gene Function
Arabidopsis	*AtMYC2* (*At1g32640*)	54.01	Activate terpene synthesase
Tobacco	Transcription factor MYC2-like	57.62	Activation of JA responses
Tomato	*SlMYC2*	57.56	Methyl jasmonate inducer
Grape vine	Predicted: transcription factor MYC2	62.01	Unknown (Genome data) *
Lindera	Transcription factor MYC	62.78	Accumulation mechanism of β-ocimene
Hayata	Transcription factor MYC2	65.13	Unknown (Genome data)
Lotus	Transcription factor MYC2-like	62.01	Unknown (Genome data)

Wintersweet *CpMYC2* was found to be homologous to the following plants with maximum identity: Arabidopsis (*Arabidopsis thaliana*), tobacco (*Nicotiana tabcum*), tomato (*Solanum lycopersicum*), grape vine (*Vitis vinifera*), lindera (*Lindera glauca*), hayata (*Cinnamomum micranthum*), lotus (*Nelumbo nucifera*). * Gene sequence is from submitted genome data with unknown function.

**Table 2:** Summary of the homology of wintersweet *CpbHLH13* with genes from other plants.

Plant Name	Gene	Identity %	Gene Function
Arabidopsis	Transcription factor *bHLH13*/JAM2 (*At1g01260*)	42.06	Interacts with JAZ proteins to negatively regulate jasmonate responses
Arabidopsis	Transcription factor *bHLH17*/JAM1 (*At2g46510*)	44.78	Interacts with JAZ proteins to negatively regulate jasmonate responses
Tobacco	Predicted: transcription factor bHLH13-like	60.26	Unknown (Genome data) *
Tomato	Transcription factor *bHLH13*-like	59.51	Unknown (Genome data)
Grape vine	Transcription factor *bHLH13*	62.90	Unknown (Genome data)
Hayata	Transcription factor *bHLH13*	69.77	Unknown (Genome data)
Lotus	Predicted: transcription factor *bHLH13*-like	62.46	Unknown (Genome data)

Wintersweet *CpbHLH13* was found to be homologous to the following plants with maximum identity: Arabidopsis (*Arabidopsis thaliana*), tobacco (*Nicotiana tabcum*), tomato (*Solanum lycopersicum*), grape vine (*Vitis vinifera*), hayata (*Cinnamomum micranthum*), lotus (*Nelumbo nucifera*). * Gene sequence is from submitted genome data with unknown function.

**Table 3:** Primers used for PCR for homozygous T-DNA insertion lines.

Primer Name	Primer Sequence (5′–3′)	TM C⁰
Salk_083483 LP	TGGTTTTTCTTGGTTTCGATG	60
Salk_083483 RP	CTCTAATCATTGCGTCCCAAC	
LBb1.3	ATTTTGCCGATTTCGGAAC	60

**Table 4:** Primers for qRT-PCR analysis.

Primer Name	Primer Sequence (5′–3′)	TM C⁰
*CpMYC2q-F*	TCCAGTCCAACAAGAAGAACCACCC	63.5
*CpMYC2q-R*	CTATCTGTTGCCAATTTTCTCCGCC	
*CpbHLH13q-F*	CCTTTGGACACCCACCCG	60.5
*CpbHLH13q-R*	CTATAATGAGTTTGATTCACGACTAAATGC	
*RPL8-F*	ACATTGCCGACCATGAGATTG	59
*RPL8-R*	CACTTGCCCGAGTTACCTTT	
*CpTublin-F*	GTGCATCTCTATCCACATCG	60
*CpTublin-R*	CAAGCTTCCTTATGCGATCC	
*AtCACS-F*	TCGATTGCTTGGTTTGGAAGAT	60
*AtCACS-R*	GCACTTAGCGTGGACTCTGTTTGATC	
*AtTubulin-F*	TCAAGAGGTTCTCAGCAGTA	59
*AtTubulin-R*	TCACCTTCTTCATCCGCAGTT	
*NtNADH-F*	TTGGTGGATCTGACCTAGTG	60
*NtNADH-R*	ATGGTGTGAAAGAGCGTTCG	
*NtCPN60-2-F*	ATGGCACTCTTGATGGGTTC	60
*NtCPN60-2-R*	AGCACTAGGCATTGCCATTG	
*35S-F*	ACGCACAATCCCACTATCCT	59
*35S-R*	TGCTCAACACATGAGCGAAAC	
*At1g61680-F*	ATGATCGATGTCATTCAAAGT	52
*At1g61680-R*	TTAAATGTTTGAGACATTTCTC	
*At3g25810-F*	TATATTTGATGTAATCATCG	53
*At3g25810-R*	TTGAACCATAGCAGTGAAGAG	
*At5g23960-F*	GGAACTGAGACGTTCAAAGAG	57
*At5g23960-R*	CGCTGTGAATAAGATTAGTGC	
*At5g44630-F*	TGGAGGAAAATATAGTGATAT	56
*At5g44630-R*	CGGTGCTGAGGTATGTGAAGA	

**Table 5:** Primers of the candidate genes used for PCR amplification.

Primer Name	Primer Sequence (5′–3′)	TM C⁰
*CpMYC2*-*F*	CGGGGTACCATGAATCTCTGGTCCGACGA	63.5
*CpMYC2-R*	CTAGTCTAGACTATCTGTTGCCAATTTTCTCC	
*CpbHLH13-F*	CGGGGTACCATGAAATCGGAGATTGGAA	60.5
*CpbHLH13-R*	CTAGTCTAGACTATAATGAGTTTGATTCACGA	

Underline sequences indicate the restriction enzyme sites.

## Supplementary Materials

The following are available online at https://www.mdpi.com/2223-7747/9/6/785/s1, Figure S1: Three-dimensional structure prediction of the protein encoded by the *CpMYC2* gene. Figure S2: Three-dimensional structure prediction of the protein encoded by the *CpbHLH13* gene. Figure S3: Phylogenetic analysis of wintersweet *CpMYC2* with the 158 arabidopsis *bHLH* transcription factor gene family. Figure S4: Phylogenetic analysis of wintersweet *CpbHLH13* with the arabidopsis *bHLH* transcription factor gene family. Figure S5: Phylogenetic analysis of *Chimonanthus praecox CpMYC2* with different plant species. Figure S6: Phylogenetic analysis of *Chimonanthus praecox CpbHLH13* with different plant species. Figure S7: Expression of the *CpMYC2* and *CpbHLH13* genes in plants. Figure S8: Partial diagram of the pCAMBIA 2300S transformation vector with *CpMYC2* and *CpbHLH13* genes. Figure S9: Standard curve of linalool and caryophyllene. Sequence S1: Nucleotide sequence of the *CpMYC2* candidate gene. Sequence S2: Nucleotide sequence of the *CpbHLH13* candidate gene.
