# Supplementary material for: Molecular Cloning and Functional Characterization of CpMYC2 and CpBHLH13 Transcription Factors from Wintersweet (Chimonanthus praecox L.)
Source: Plants (Basel). 2020 Jun 23;9(6):785. doi: 10.3390/plants9060785 (PMC7356763; doi:10.3390/plants9060785)
Supplement: Supplementary file 1 [file plants-09-00785-s001.zip › Supplementry data revised 21 June 20/Supplementary data file 21 June.docx]

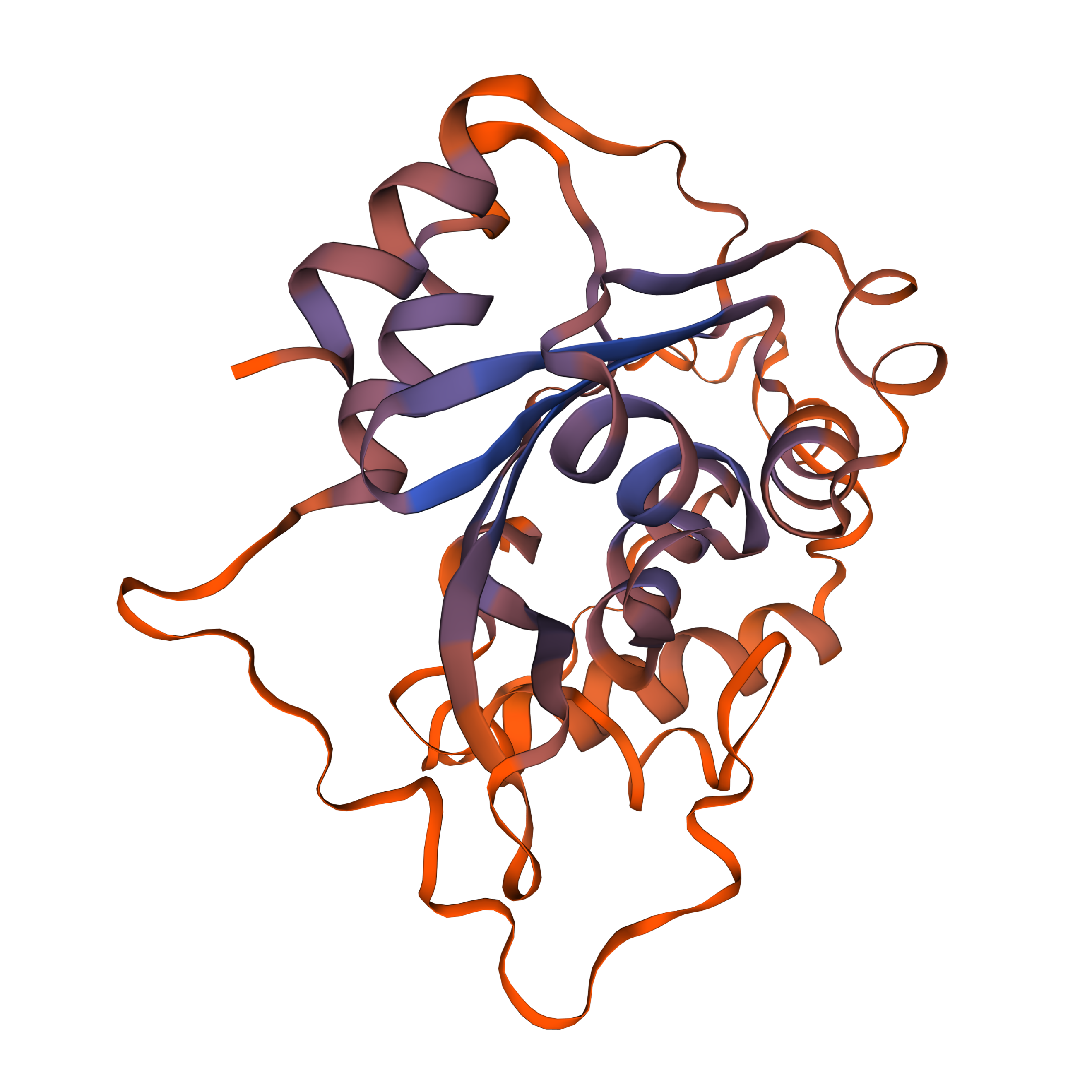


**Supplementary Fig 1-** Three dimensional structure prediction of the protein encoded by *CpMYC2* Gene.


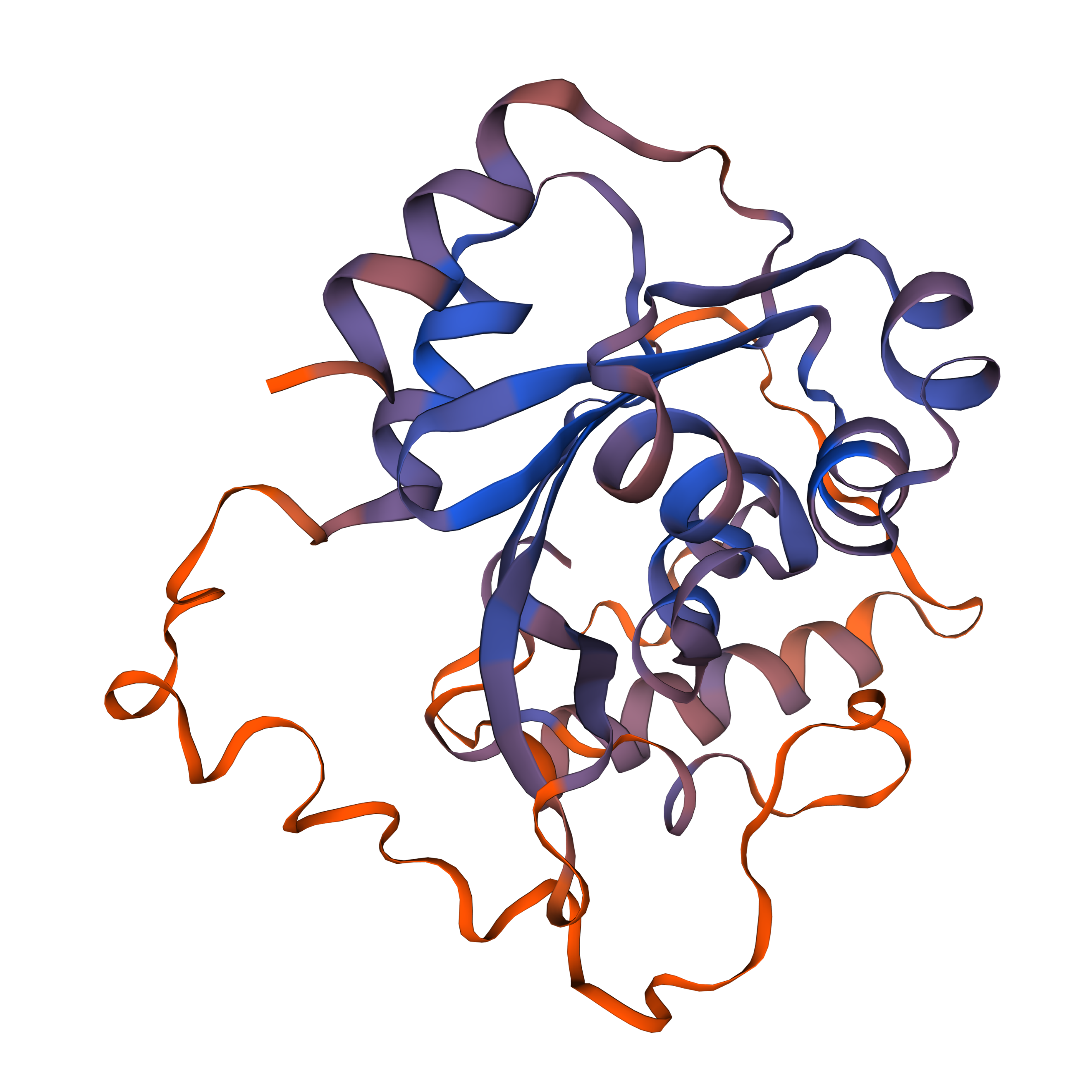

**Supplementary Fig 2-** Three dimensional structure prediction of the protein encoded by *CpbHLH13* gene.


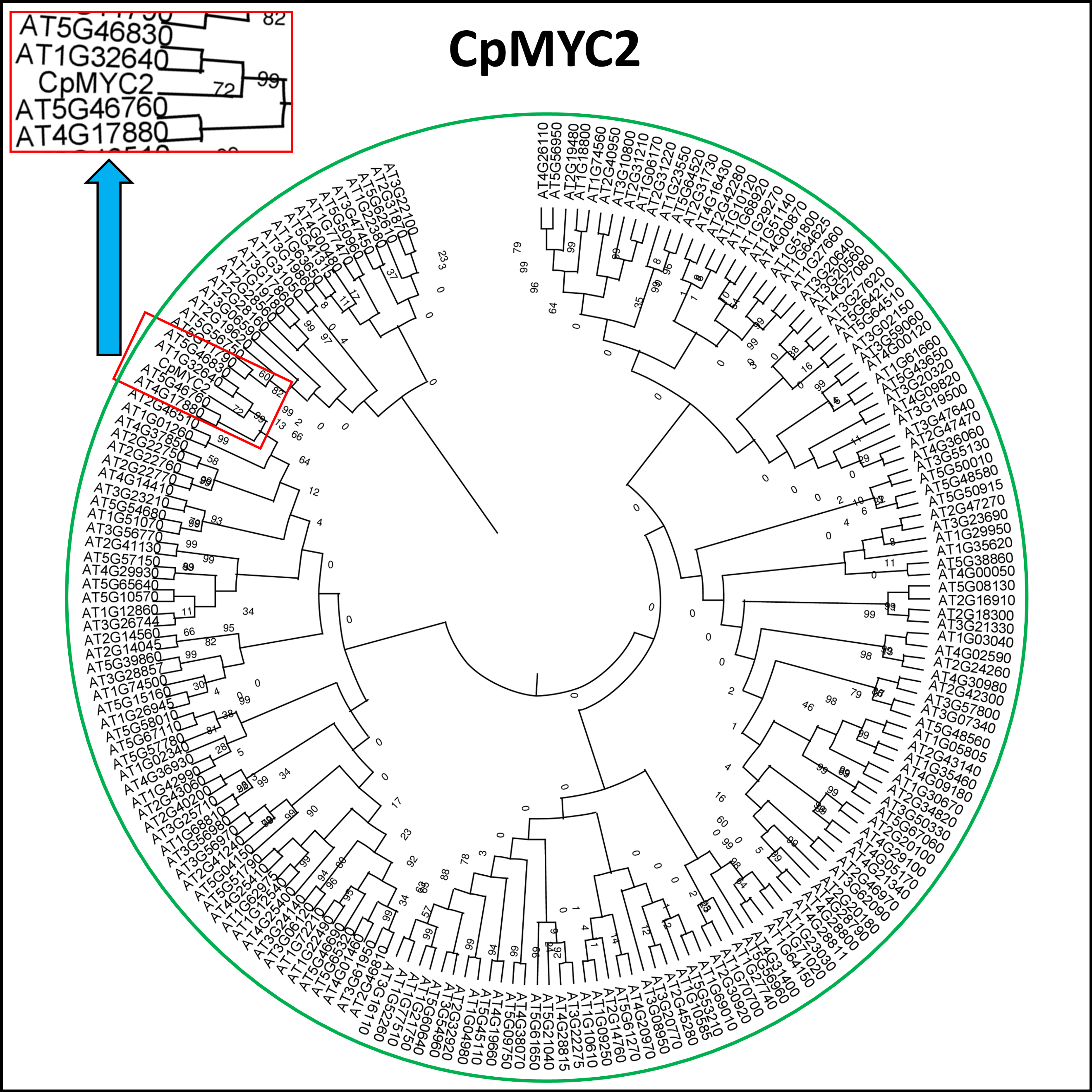


**Supplementary Fig 3-** Phylogenetic analysis of wintersweet *CpMYC2* with arabidopsis *bHLH* transcription factor gene family. The red box indicates the close homologous *bHLH* of arabidopsis with *CpMYC2.*


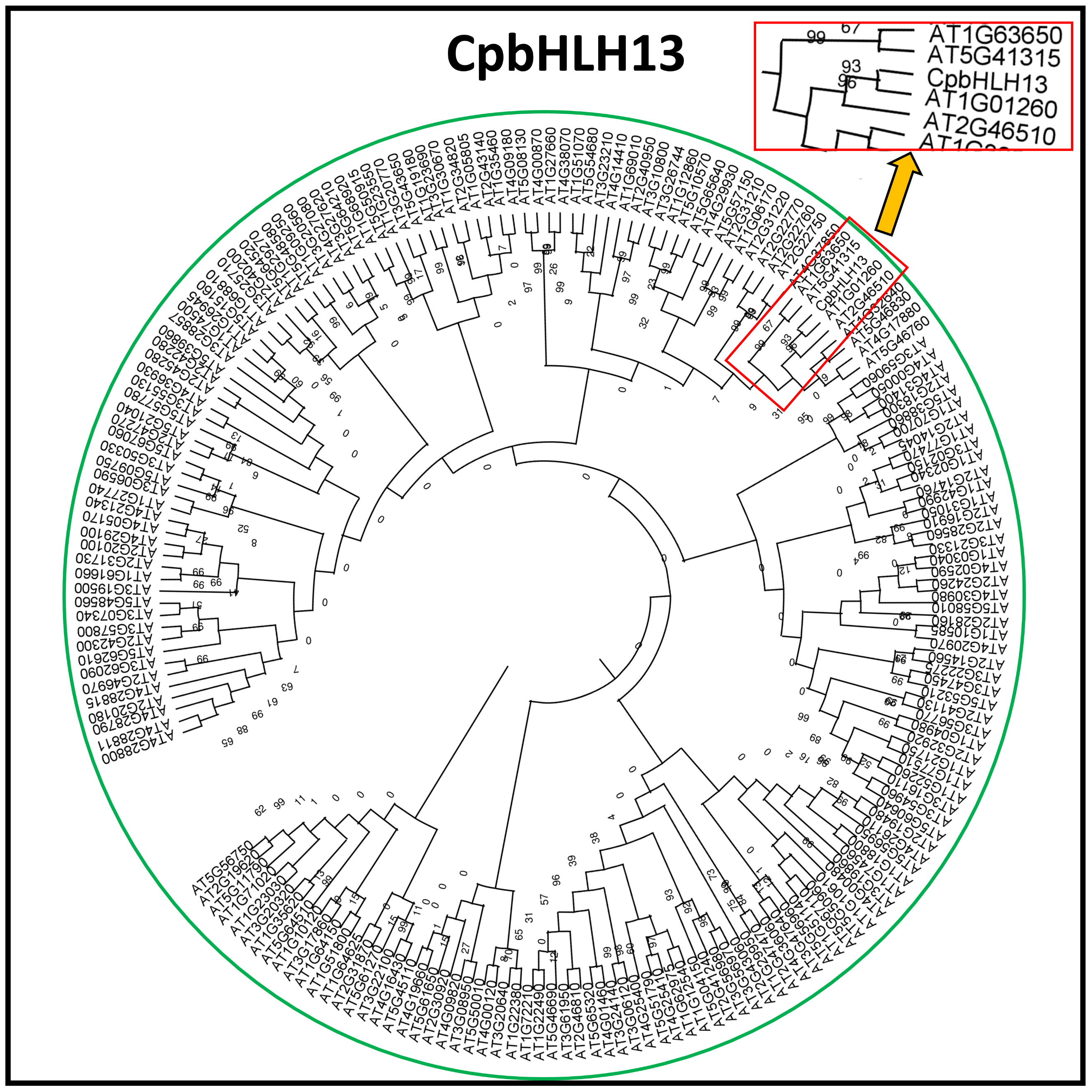


**Supplementary Fig 4-** Phylogenetic analysis of wintersweet *CpbHLH13* with the arabidopsis *bHLH* transcription factor gene family. The red box indicates the close homologous *bHLH* of arabidopsis with *CpbHLH13.*


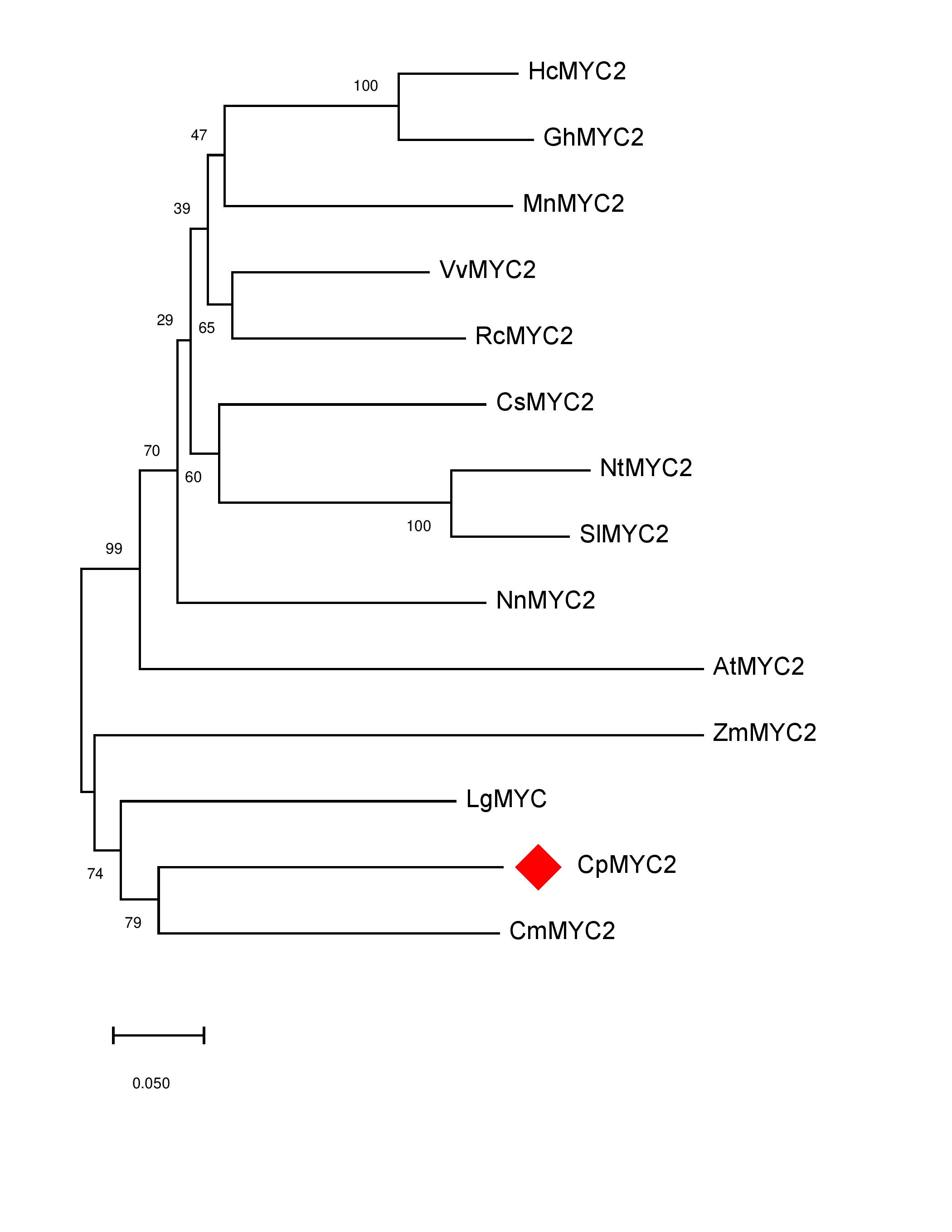


**Supplementary Fig 5-** Phylogenetic analysis *of Chimonanthus praecox* *CpMYC2* with different plant species. The phylogenetic tree was constructed based on the amino acid sequences of *CpMYC2* and the MYC2 transcription factors from the plants, such as *Hibiscus cannabinus* HcMYC2 (AYU74917.1), *Gossypium hirsutum* GhMYC2 (NP_001314093.1), *Morus notabilis* MnMYC2 (XP_010104300.1), *Vitis vinifera* VvMYC2 (XP_002280253.2), *Ricinus communis* RcMYC2 (XP_002519814.1), *Camellia sinensis* CsMYC2 (XP_028062859.1), *Nicotiana tabacum* NtMYC2 (XP_016500373.1), *Solanum lycopersicum* SlMYC2 (NP_001311412.1), *Nelumbo nucifera* NnMYC2 (XP_010275210.1), *Arabidopsis thaliana* AtMYC2 (At1g32640), *Zea mays* ZmMYC2 (PWZ55921.1), *Lindera glauca* LgMYC (ALE71301.1) and *Cinnamomum micranthum* CmMYC2 (RWR86802.1). The sequences were aligned using the CLUSTALW program and the phylogenetic tree was generated with the neighbor binding method with 1000 repetitions of the start test, p-distance using the MEGA X software.


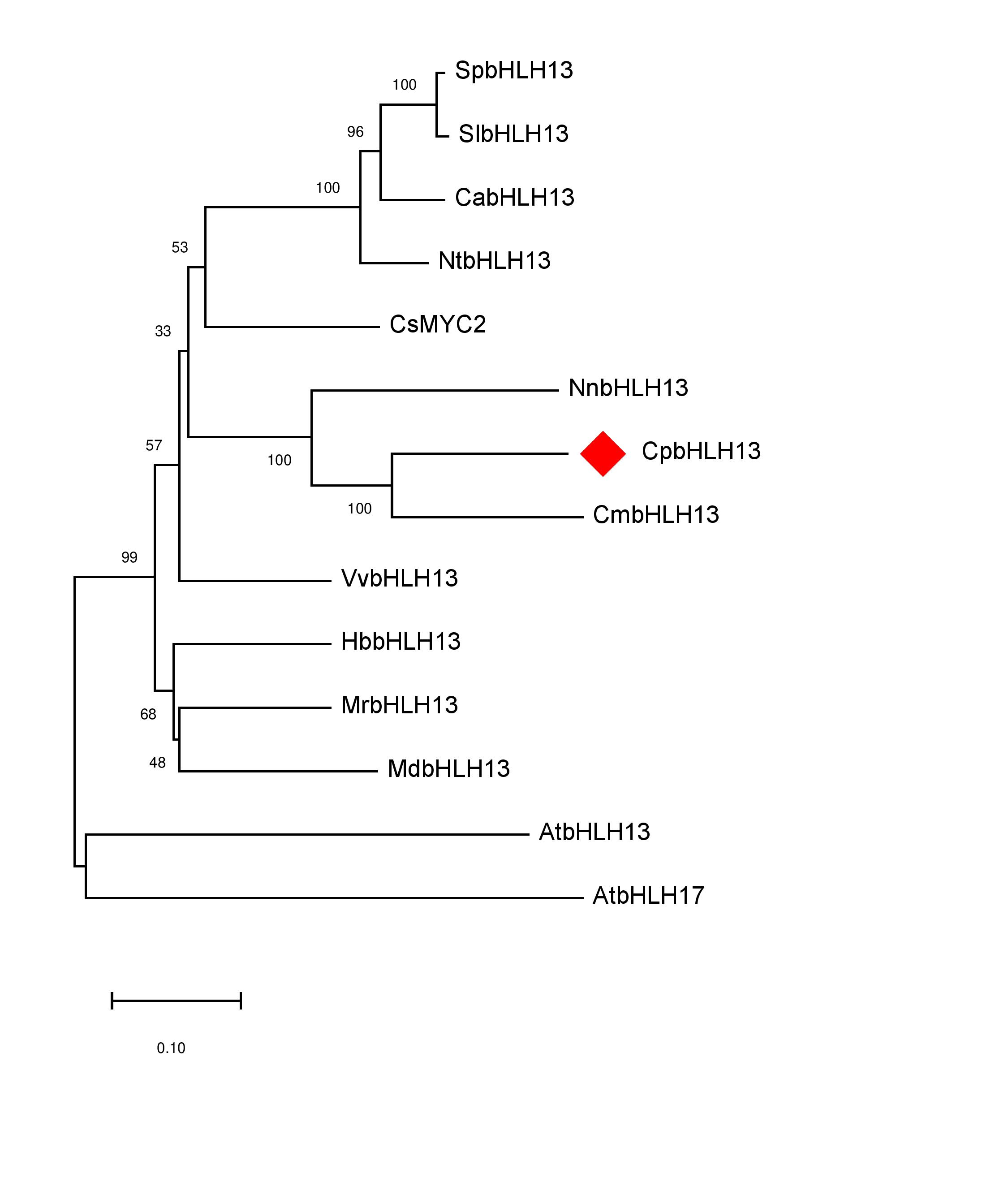


**Supplementary Fig 6-** Phylogenetic analysis *of Chimonanthus praecox* *CpbHLH13* with different plant species. The phylogenetic tree was constructed based on the amino acid sequences of *CpbHLH 13* and the *bHLH* transcription factors from the plants, such as *Solanum pennellii* SpbHLH13 (XP_015063116.1), *Solanum lycopersicum* SlbHLH13 (XP_004229991.1), *Capsicum annuum* CabHLH13 (KAF3659155.1), *Nicotiana tabacum* NtbHLH13 (XP_016459177.1), *Camellia sinensis* CsbHLH13 (ANB66342.1), *Nelumbo nucifera* NnbHLH13 (XP_010242396.1), *Cinnamomum micranthum* CmbHLH13 (RWR96436.1), *Vitis vinifera* VvbHLH13 (RVW95465.1), *Hevea brasiliensis* HbbHLH13 (XP_021664882.1), *Morella rubra* MrHLH13 (KAB1219634.1), *Malus domestica* MdbHLH13 (XP_028955372.1), *Arabidopsis thaliana* AtbHLH13 *(*AT1G01260) and *Arabidopsis thaliana* AtbHLH17 (At2g46510). The sequences were aligned using the ClustalW program and the phylogenetic tree was generated with the neighbor binding method with 1000 repetitions of the start test, p-distance using the MEGA X software.

**
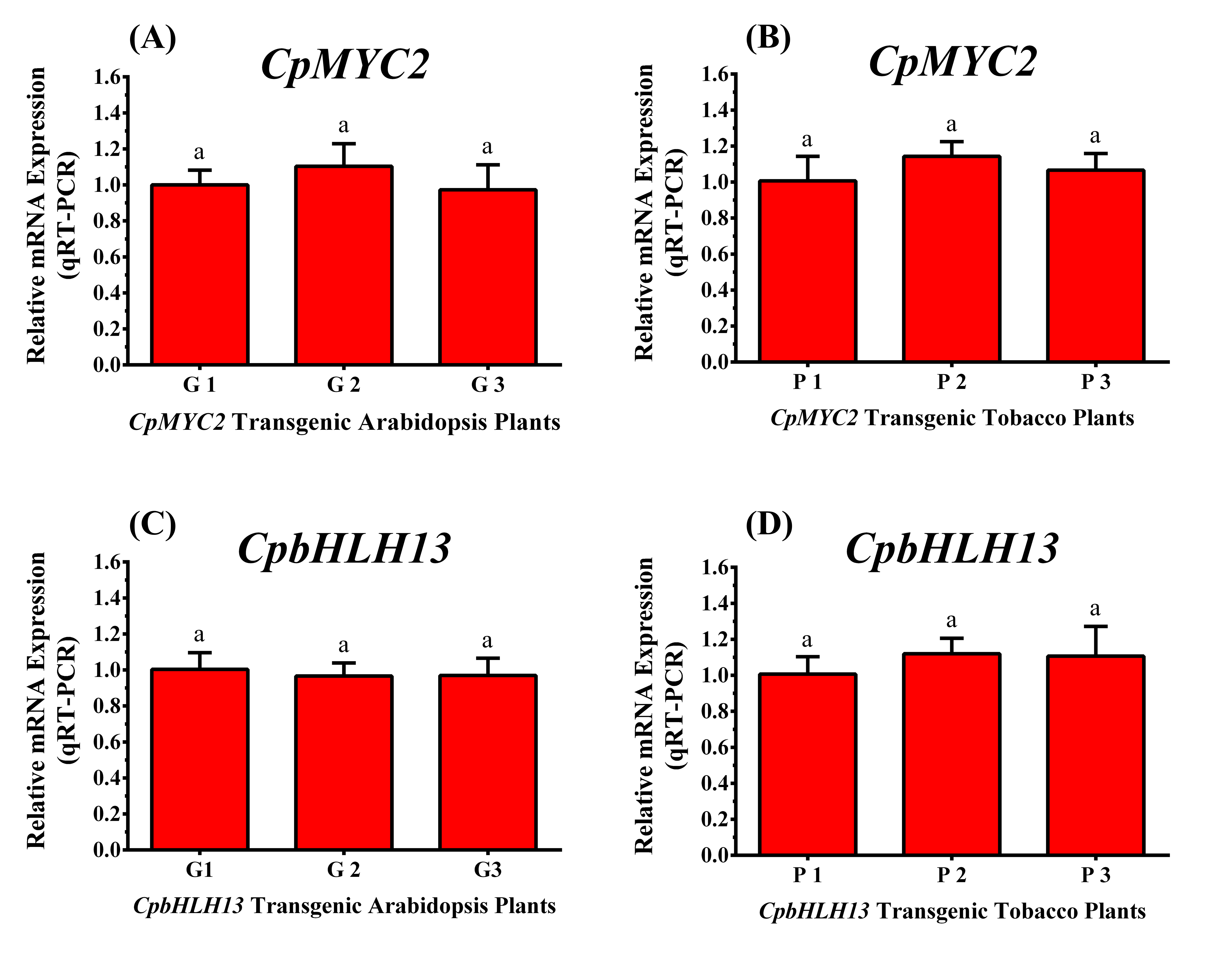
**

**Supplementary Fig 7-** Expression of *CpMYC2* genes in transgenic **(A)** Arabidopsis and **(B)** Tobacco and expression of *CpbHLH13* genes in **(C)** Arabidopsis and **(D)** Tobacco by qRT-PCR. P (1-3) represents the number of the transgenic tobacco plant and G (1-3) indicates the group number of arabidopis plants. The different letters on the bars show significance between the treatments by using least significant difference (LSD) at *p*<0.05.

**
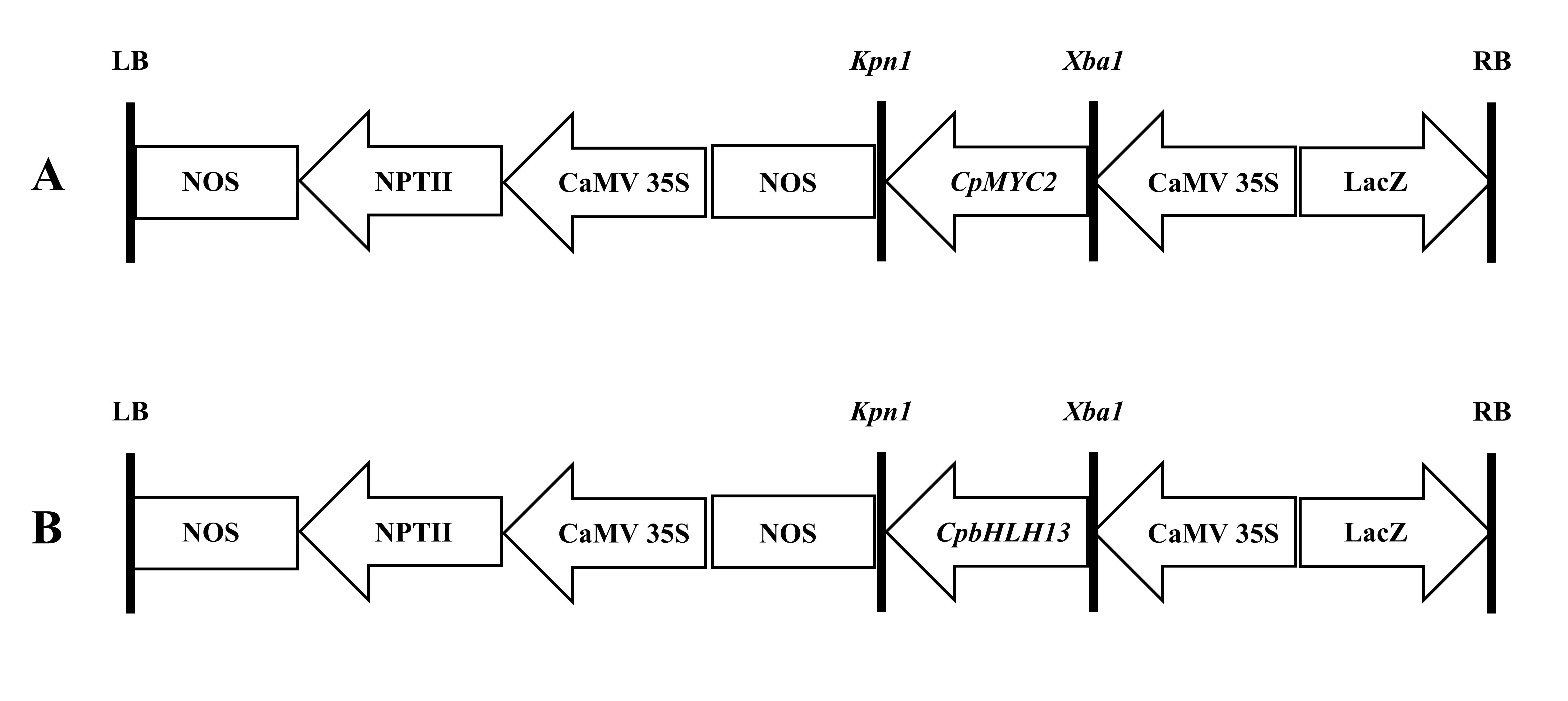
**

**Supplementary Fig 8-** Partial diagram of the pCAMBIA 2300S transformation vector with (A) *CpMYC2* and (B) *CpbHLH13* genes under the control of CaMV 35S promoter, followed by NOS terminator. The neomycin phosphotransferase gene (NPT II) is driven by CaMV 35S promoter as a selectable.

**
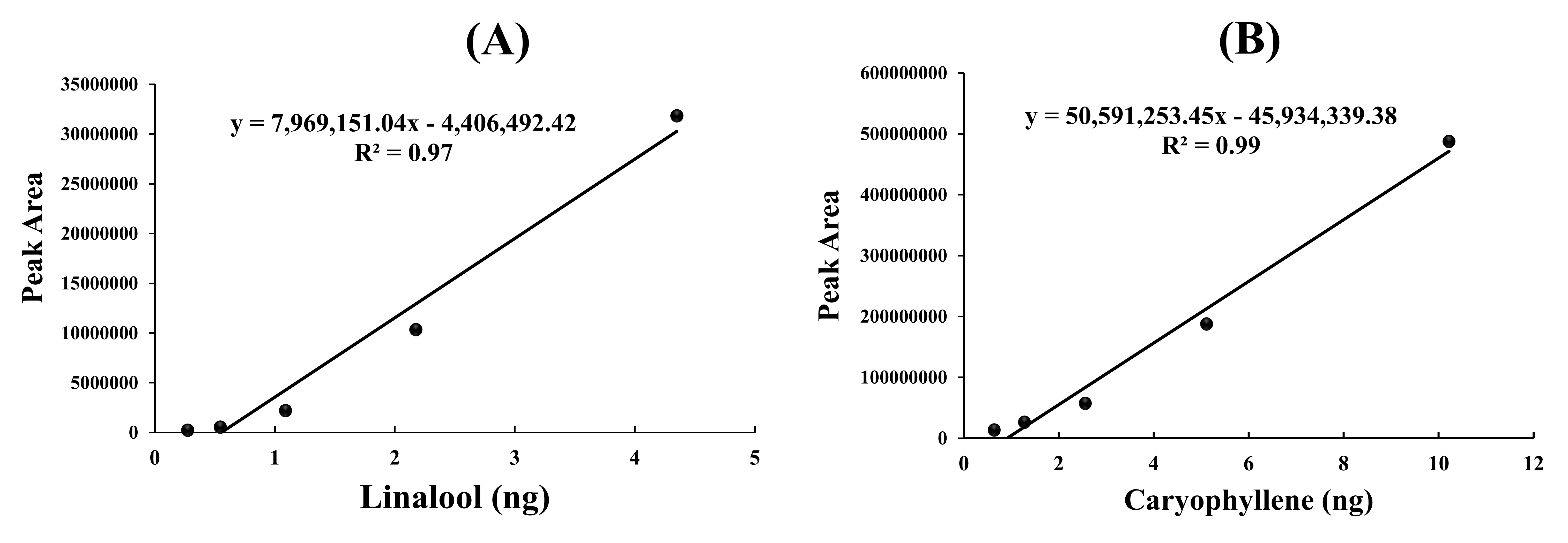
**

**Supplementary Fig 9-** The standard curve of **(A)** linalool and **(B)** Caryophyllene. The curve was generated by three repeats. X stands for the content of Linalool (ng) and Y for the peak area.

**Supplementary Sequence S1.** Nucleotide sequence of *CpMYC2* candidate gene.

ATGAATCTCTGGTCCGACGACAACGCTTCGGTCATGGAAGCCTTCATGACTTCAGACCTCCACTCCTTCGGCTGGGGCGCCCCATCTTCGTCCGCCTCCGCCCAACCCACGGCTGCGATGGCGTCCACGACAGATCCTGGATCCAGGGGCGTCAATCAGGAGACTCTGCAGCAGCGGCTACAGGCTCTGATAGAGGGTGCACGGGAAAGCTGGACCTACGCCATCTTCTGGCAGTCGTCCGTTGACGTTGGGGGTTCCACCCTCCTCGGCTGGGGTGACGGCTACTACAAGGGTGAGGAGGACCGCCGCCGCCGCATGGCCGCAGCCTCGGCTGCCGATCAGGAGCACAGGAAGCGTGTTCTGCGGGAGCTCAACTCGCTCGTCTCCGGCGCATCTGCCTCCGACGATGCGGTCGACGACGAGGTGACCGACACCGAGTGGTTCTTCCTCGTCTCCATGACCCAGCTCTTCGTCGACGGCACTGCGCTCCCCGGACAGTCCTTTTTCTCGGCCTCCCCCATTTGGGTGGCTGGTGCCAACCAGCTCGCCACCTCCTCCTGCGAGCGGGCGAGGCAGGCGCAGGTGTTTGGGCTCCAGACCATGGTCTGCATCCCCTTCGGCAACGGCGTCGTCGAGCTAGGCTCCACCGATCCCATCATTAATAATTCTGATCTTATGAACAAGGCACGGGTACTCTTTAATTTCGACGCCGGCGGGTGGCCTGACCAGGCTGAAAACGACCCTTCGGCGCTCTGGATCACGGAGCCCTCTTCTGCCACCGGGGTCGAGATTAAGGACTCCGGAAGCACTGCTGTGGCAGAGGCCTCCTTGTCAAAATCGATCCACTTCGACAATCAAAGCACGAGCGGCTTGACGGAGAACCATTCGCAGCATCAGCAGAATCACAACCACGAAAAGCAAAATTATCACCACGGTAATTTCTTCAGTAAAGAGATGAATTTCTCCGAATTGGGATTCGAAGGCAGTAGAAGCTCGCAGCCTTGCAAGCTGGAATCGGGCGAGATCTTGAATTTTGGAGACAGCAGGAGGAATTCCAGCTCTAATGGAGGAACTCTTTTCTCCCAATTTCAGCAGATTCCTTCTGTTGACGACGAGAAGAACAGGAAGAGGTCAAGGAACAGCAAGGACGAGGGGATGCTCTCATTTGCATCGGCGGTTGTCTTGCCCTCTTCCGGGATGGTGAAATCCGGGGATTCCGACCATTCTGATCTTGAAGCTTCAGTCCGAGAGGCAGAGAGCAGCAGAGTAGTTGCCGACACCGAGAAGCGCCCGAGAAAGCGGGGACGCAAGCCCGCAAATGGTCGGGAGGAGCCGCTGAACCACGTAGAGGCAGAACGGCAGCGGAGGGAGAAACTCAACCAGAGATTCTACGCTCTCCGAGCCGTCGTCCCCAACGTGTCCAAGATGGACAAGGCCTCCCTCCTTGGCGACGCCATCGCCTACATCAACGAACTCAAGTCCAAGATGCAGACAATGGAGTCAGAGAAGGATCAATTACATGCCCAAATAGATGTCCTGAAGAAGGACCTCAAGATCTCCATCTCGAATTCCGATTTAAACTCCAATCCAAATTCAAATTCCGGGTCTGCGGCTCCACTGAGTGCGGTGGAGATGGATGTGAAGATACTGGGGAGGGAAGCAATGATCCGAATCCAGTCCAACAAGAAGAACCACCCGGCGGCACGTCTGATGTCGGCGCTGAAGGAGCTGGATCTGGAGCTGCACTACGCAAGTGTGTCGGTGGTAAAGGATCTGATGATCCAGCAGGCGACGGTTAAGATGTCAAGCCGGATCTACAGTCAGGAGCAGCTGAGCTCCGCCCTCATGGCCAAAATAGGGGCGGAGAAAATTGGCAACAGATAG

**Supplementary Sequence S2.** Nucleotide sequence of *CpbHLH13* candidate gene.

ATGAAATCGGAGATTGGAATCGGAAAATTTTGGAGCGATGAAGACAAGGGGATGGTGATGGCTATCATGGGCCGGGTCGCTTTCGATTATTTGACGGCGAGCAATGTCTCCTCCGAGGGGTTGCTCTCCGTTGTACCTGGCGGCGATGGAAATTTGCAGAACAAGTTAATGGAGCTCGTCGAGGGATCGAATCCCTTCAATTTGGGTTGGAATTACGCCATCTTTTGGCAGATTTCAAGATCCAAATCCGGAGATTTGGTTCTTGGTTGGGGAGACGGGTATTGCAGGGAAGAGTCCGAGAGCCGATTTAATCGGGTCTCGGATGCCCGATTAGAGGACGACGCCACACAGCAGAAGATGCGCAAACGGGTCCTCCAAAAGCTCCATACCTTGTTCGGAGGAACCGACGAGGAGAGTGCTTTCGGGTTGGATCGGTTATCCGATACGGAAATGTTCTTTCTCACGTCCATGTACTTCTCGTTTCCTCGTGGGGAAGGCGGGCCAGGTCGGGTATTTGGGTCCGGGCAGCATCTTTGGCTGCCGGATGCACTCAATTCTCCCGGTGATTACTGCATCCGTTCGTTCCTCGCGAGGTCTGCCAGGATCCAGACTGCGGTGCTGGTTCCTATCGATACTGGTGTGATTGAATTGGGTTCATTGAGGTCGATTCCAGAGAATTTTGATGTTGTTCAAAGGATAAAGTCTGTCTTTTCAAGGACGGCCCAGATAACGCCGGGGAAGGATGAGAATTGCCGGGTTTCAGGATTAGGGTTTGGTGGGGGTGATCGGGTGGAGGAGTGCCCGACGAAGATTTTTGGTCAGGATCTGAATCAAGGTCGGTCACAGATGAAGGTGGAGGAGAGGCCATGTGAAATGTACCCTGGCGGAGCTGGAGTGGGCAGTAACCGGAGCCCGTTTCCCAGAAAGGGTTTTCATGGACTGGCCTGGAATCAGACCCATGTTGCTAACAATGCCCAGAAATTCAATAATGGCGTTTTGATGATTGGTAATGATGGTGATTCTGCGGCAGCATATCGAGCGTATGGTCACTCTAATGGAGTTCGAGATGATCCCCGAATGACCCAATTTCAACCTCAGCAGCAACAAAGGCAAATTGATTTCTCTGGGGCGCCGTCAAGGGGTTCAGTAATCAACCGTCCAGGTGCAGTTGAATCCGAGCACTCAGATATTGAGGCTTCATGCAAGGAAGACCGAGCAGGGCTAGAAGATGATCGAAAACCCAGAAAGAGAGGCCGAAAGCCAGCAAATGGAAGAGAGGAACCCTTGAACCATGTTGAAGCTGAGCGCCAGCGACGCGAGAAGCTCAATCAGCGTTTCTATGCATTGAGGGCAGTAGTGCCCAACATCTCGAAAATGGACAAGGCGTCATTGCTCGGAGACGCTATAGCTTATATCACTGAGCTTCAGAAGAAGCTCAAAGAGATGGAATCTGAAAGAGAGAGATTTGGATCCACATCCAAAGATGCAACCTCTGAAGGAAATTCAGTCGAGAATCTGAAATCCATTCCTGCACCCGATATCGATATCCAAGCAGTTAAAGATGAGGTTATTGTTCGAGTGAGCTGCCCTTTGGACACCCACCCGGTTTCGAAAGTTATACATGCATTCAAAGAGGCGCAAATCTCTGTCCTTGAGTCGAAGATCGCTGCTGGTAATGATACCGTTTTCCACACTTTCGTCCTTAAGTCACAAGGATCCGAGCAGCTTACTAAGGAGAAGCTGATTGCTGCATTTAGTCGTGAATCAAACTCATTATAG
