# Supplementary material for: Molecular Cloning and Functional Characterization of CpMYC2 and CpBHLH13 Transcription Factors from Wintersweet (Chimonanthus praecox L.)
Source: Plants (Basel). 2020 Jun 23;9(6):785. doi: 10.3390/plants9060785 (PMC7356763; doi:10.3390/plants9060785)
Supplement: Supplementary file 1 [file plants-09-00785-s001.zip › Supplementry data revised 21 June 20/Supplementary Final Figures 21 June/Sequence S2.docx]

**Supplementary Sequence S2.** Nucleotide sequence of *CpbHLH13* candidate gene.

ATGAAATCGGAGATTGGAATCGGAAAATTTTGGAGCGATGAAGACAAGGGGATGGTGATGGCTATCATGGGCCGGGTCGCTTTCGATTATTTGACGGCGAGCAATGTCTCCTCCGAGGGGTTGCTCTCCGTTGTACCTGGCGGCGATGGAAATTTGCAGAACAAGTTAATGGAGCTCGTCGAGGGATCGAATCCCTTCAATTTGGGTTGGAATTACGCCATCTTTTGGCAGATTTCAAGATCCAAATCCGGAGATTTGGTTCTTGGTTGGGGAGACGGGTATTGCAGGGAAGAGTCCGAGAGCCGATTTAATCGGGTCTCGGATGCCCGATTAGAGGACGACGCCACACAGCAGAAGATGCGCAAACGGGTCCTCCAAAAGCTCCATACCTTGTTCGGAGGAACCGACGAGGAGAGTGCTTTCGGGTTGGATCGGTTATCCGATACGGAAATGTTCTTTCTCACGTCCATGTACTTCTCGTTTCCTCGTGGGGAAGGCGGGCCAGGTCGGGTATTTGGGTCCGGGCAGCATCTTTGGCTGCCGGATGCACTCAATTCTCCCGGTGATTACTGCATCCGTTCGTTCCTCGCGAGGTCTGCCAGGATCCAGACTGCGGTGCTGGTTCCTATCGATACTGGTGTGATTGAATTGGGTTCATTGAGGTCGATTCCAGAGAATTTTGATGTTGTTCAAAGGATAAAGTCTGTCTTTTCAAGGACGGCCCAGATAACGCCGGGGAAGGATGAGAATTGCCGGGTTTCAGGATTAGGGTTTGGTGGGGGTGATCGGGTGGAGGAGTGCCCGACGAAGATTTTTGGTCAGGATCTGAATCAAGGTCGGTCACAGATGAAGGTGGAGGAGAGGCCATGTGAAATGTACCCTGGCGGAGCTGGAGTGGGCAGTAACCGGAGCCCGTTTCCCAGAAAGGGTTTTCATGGACTGGCCTGGAATCAGACCCATGTTGCTAACAATGCCCAGAAATTCAATAATGGCGTTTTGATGATTGGTAATGATGGTGATTCTGCGGCAGCATATCGAGCGTATGGTCACTCTAATGGAGTTCGAGATGATCCCCGAATGACCCAATTTCAACCTCAGCAGCAACAAAGGCAAATTGATTTCTCTGGGGCGCCGTCAAGGGGTTCAGTAATCAACCGTCCAGGTGCAGTTGAATCCGAGCACTCAGATATTGAGGCTTCATGCAAGGAAGACCGAGCAGGGCTAGAAGATGATCGAAAACCCAGAAAGAGAGGCCGAAAGCCAGCAAATGGAAGAGAGGAACCCTTGAACCATGTTGAAGCTGAGCGCCAGCGACGCGAGAAGCTCAATCAGCGTTTCTATGCATTGAGGGCAGTAGTGCCCAACATCTCGAAAATGGACAAGGCGTCATTGCTCGGAGACGCTATAGCTTATATCACTGAGCTTCAGAAGAAGCTCAAAGAGATGGAATCTGAAAGAGAGAGATTTGGATCCACATCCAAAGATGCAACCTCTGAAGGAAATTCAGTCGAGAATCTGAAATCCATTCCTGCACCCGATATCGATATCCAAGCAGTTAAAGATGAGGTTATTGTTCGAGTGAGCTGCCCTTTGGACACCCACCCGGTTTCGAAAGTTATACATGCATTCAAAGAGGCGCAAATCTCTGTCCTTGAGTCGAAGATCGCTGCTGGTAATGATACCGTTTTCCACACTTTCGTCCTTAAGTCACAAGGATCCGAGCAGCTTACTAAGGAGAAGCTGATTGCTGCATTTAGTCGTGAATCAAACTCATTATAG
