# Supplementary figures and images for: Molecular Cloning and Functional Characterization of CpMYC2 and CpBHLH13 Transcription Factors from Wintersweet (Chimonanthus praecox L.)
Source: Plants (Basel). 2020 Jun 23;9(6):785. doi: 10.3390/plants9060785 (PMC7356763; doi:10.3390/plants9060785)

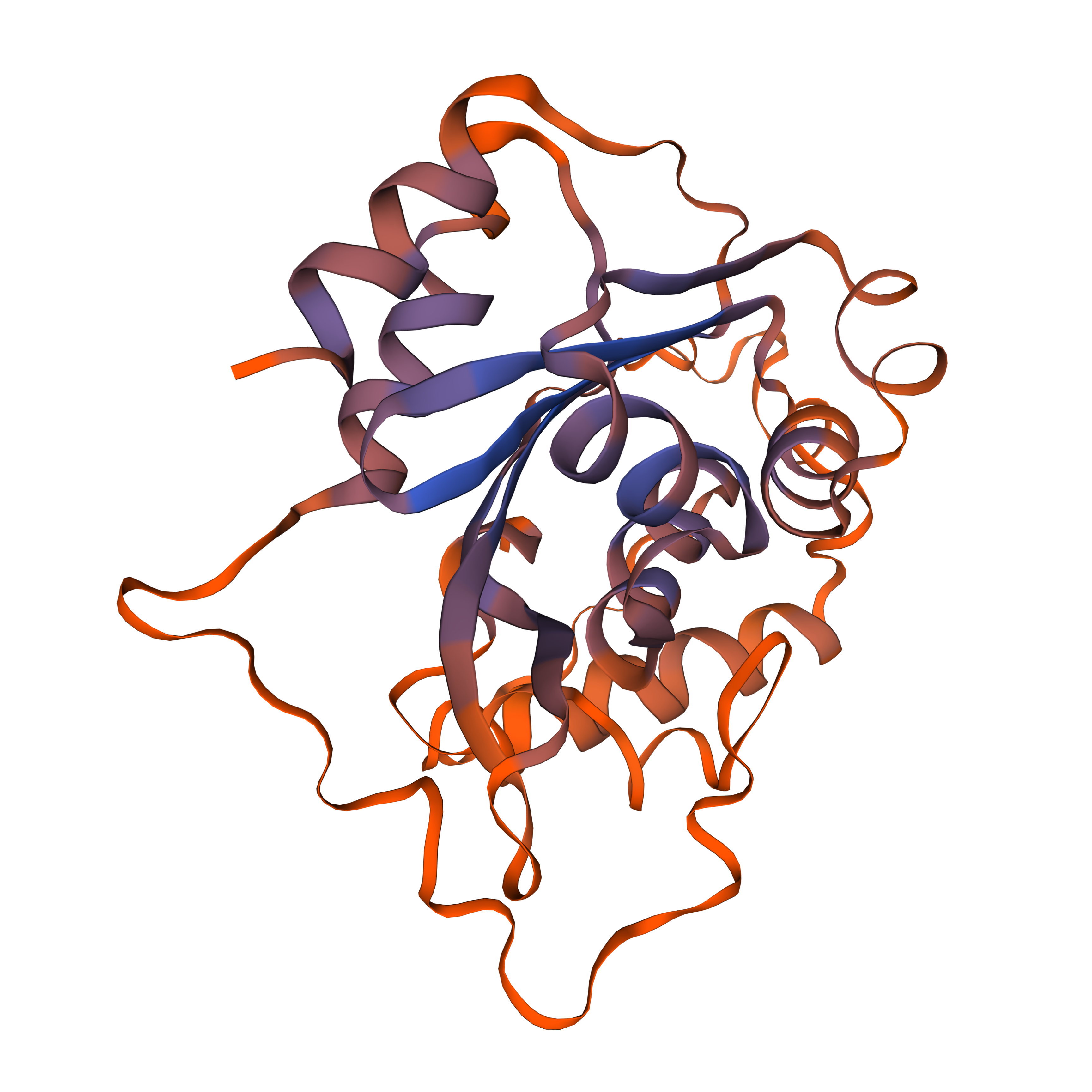

Supplement: Supplementary file 1 [file plants-09-00785-s001.zip › Supplementry data revised 21 June 20/Supplementary Final Figures 21 June/Supplementary Fig 1.tif]

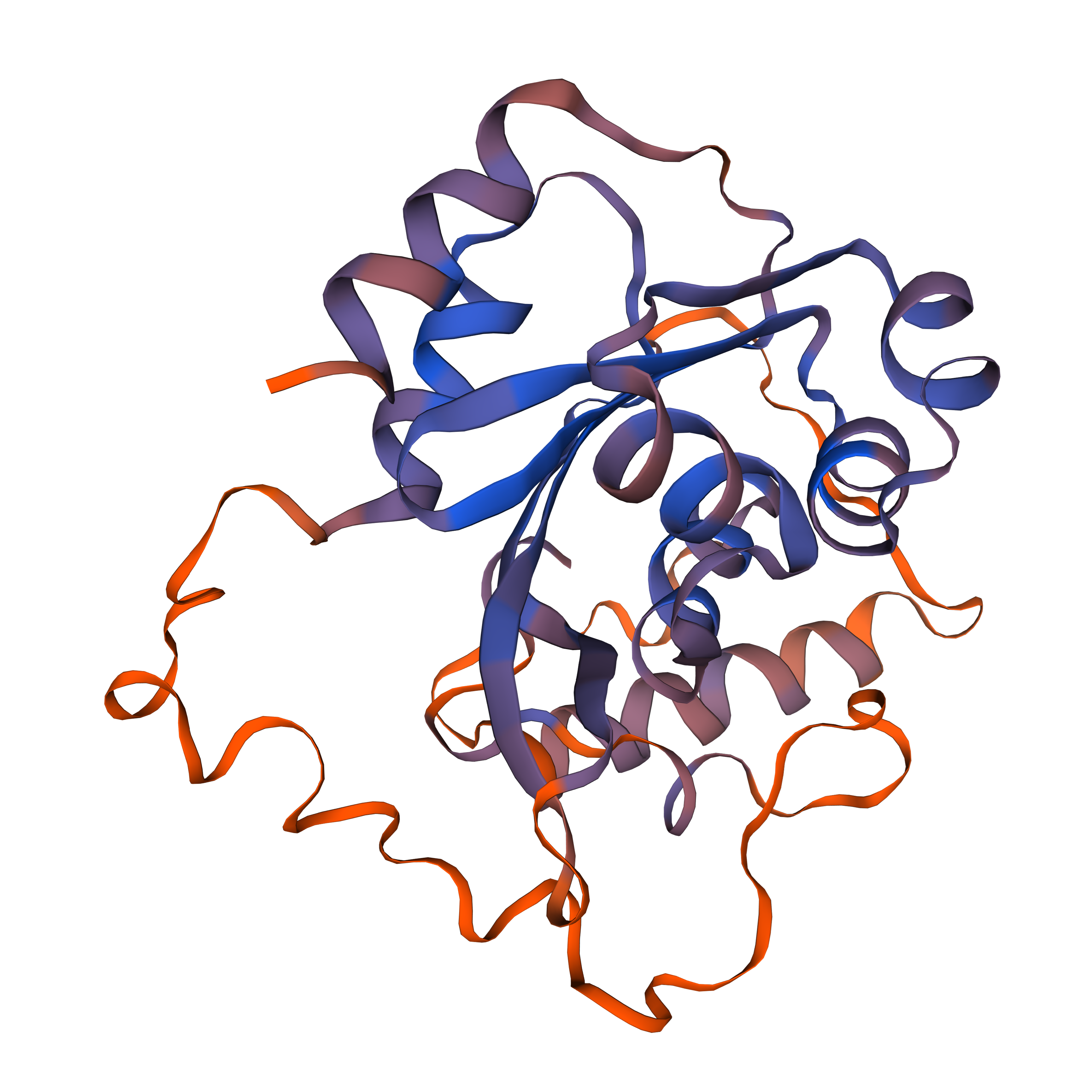

Supplement: Supplementary file 1 [file plants-09-00785-s001.zip › Supplementry data revised 21 June 20/Supplementary Final Figures 21 June/Supplementary Fig 2.tif]

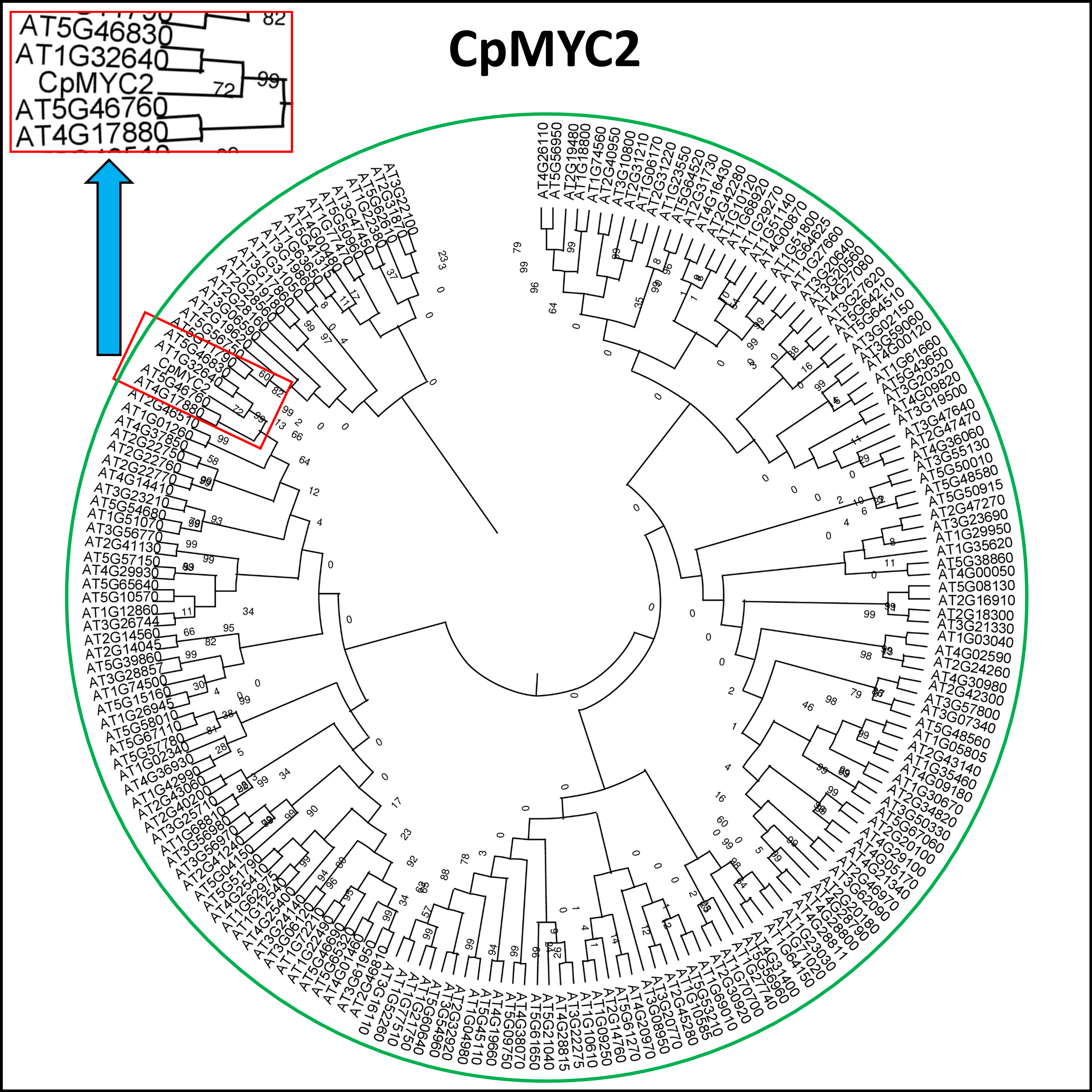

Supplement: Supplementary file 1 [file plants-09-00785-s001.zip › Supplementry data revised 21 June 20/Supplementary Final Figures 21 June/Supplementary Fig 3.tif]

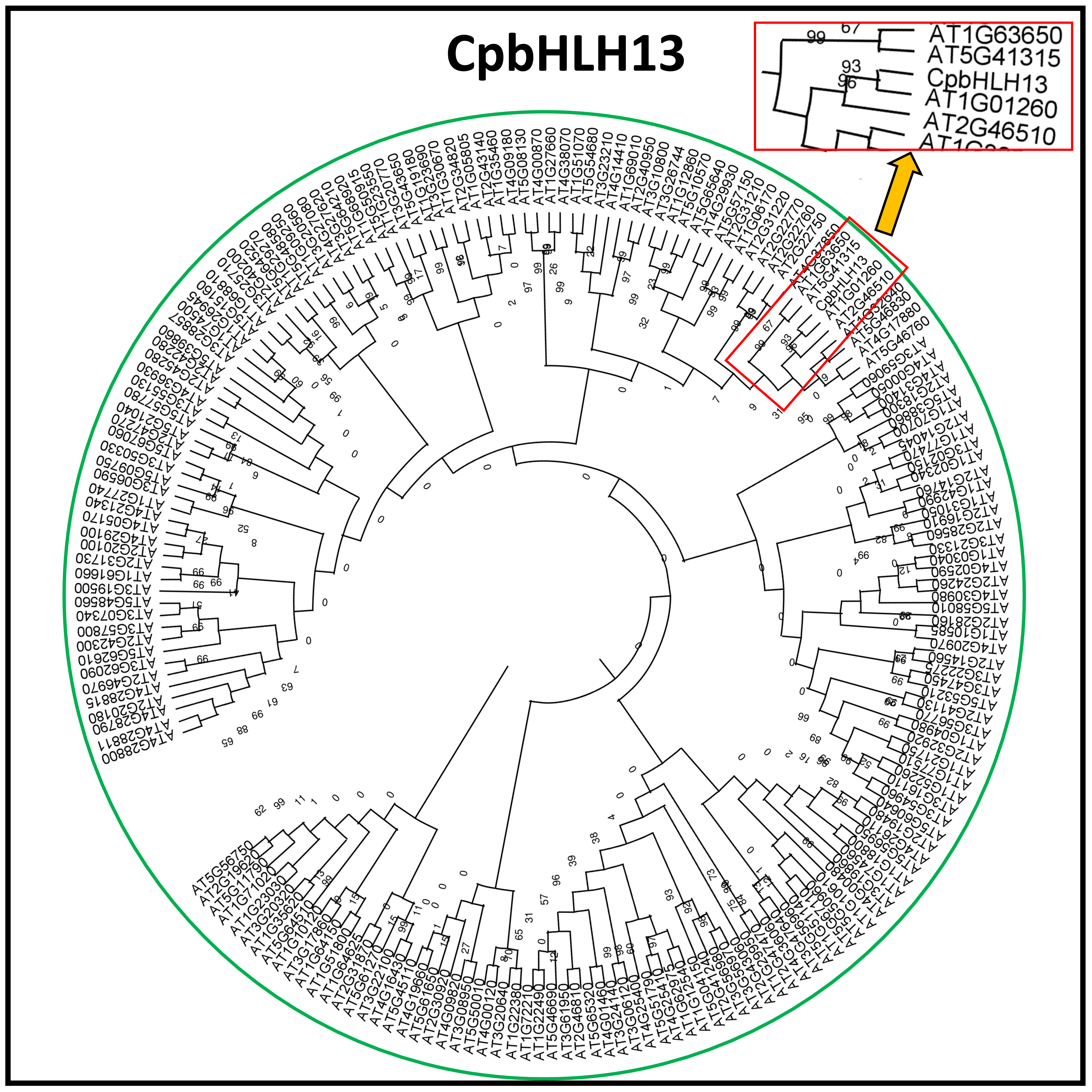

Supplement: Supplementary file 1 [file plants-09-00785-s001.zip › Supplementry data revised 21 June 20/Supplementary Final Figures 21 June/Supplementary Fig 4.tif]

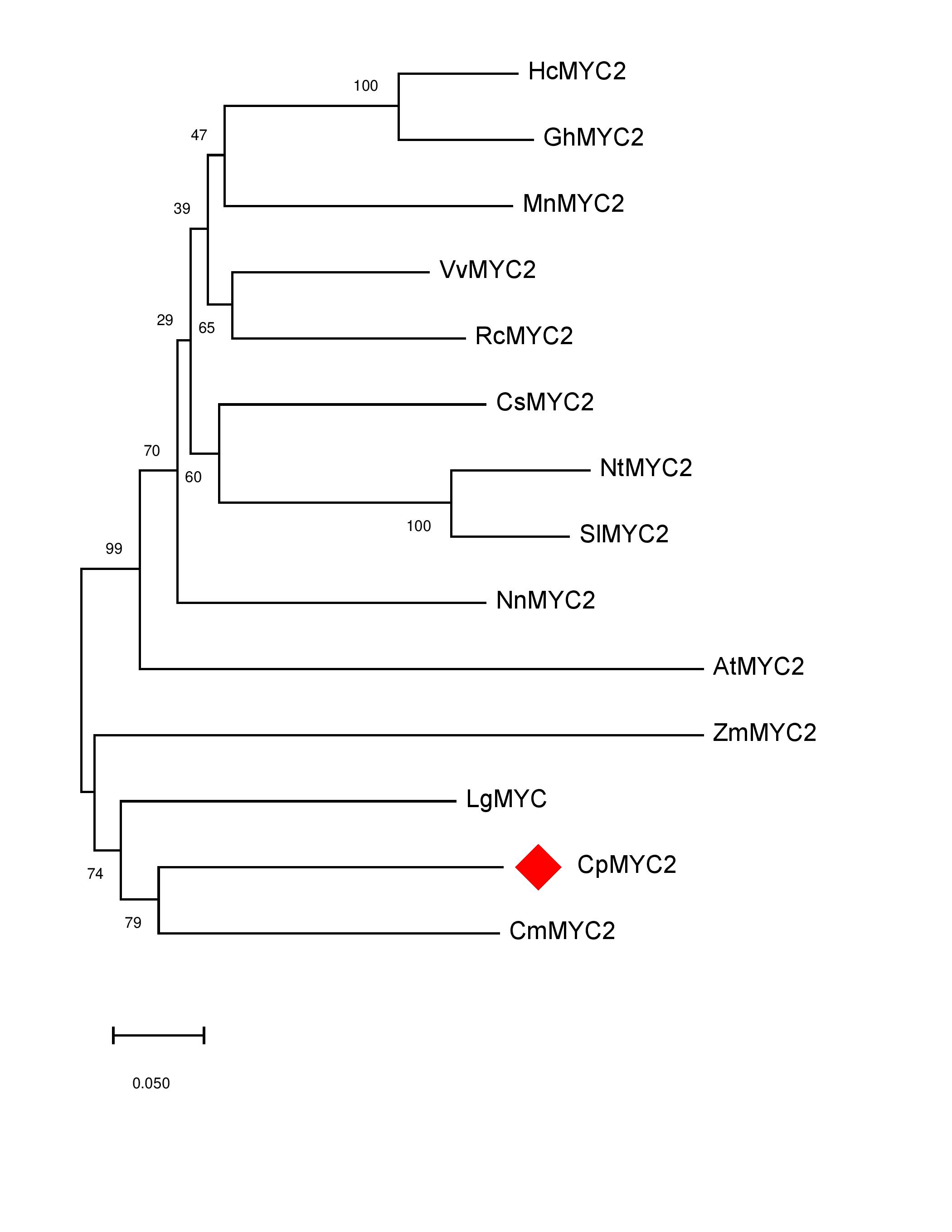

Supplement: Supplementary file 1 [file plants-09-00785-s001.zip › Supplementry data revised 21 June 20/Supplementary Final Figures 21 June/Supplementary Fig 5.jpg]

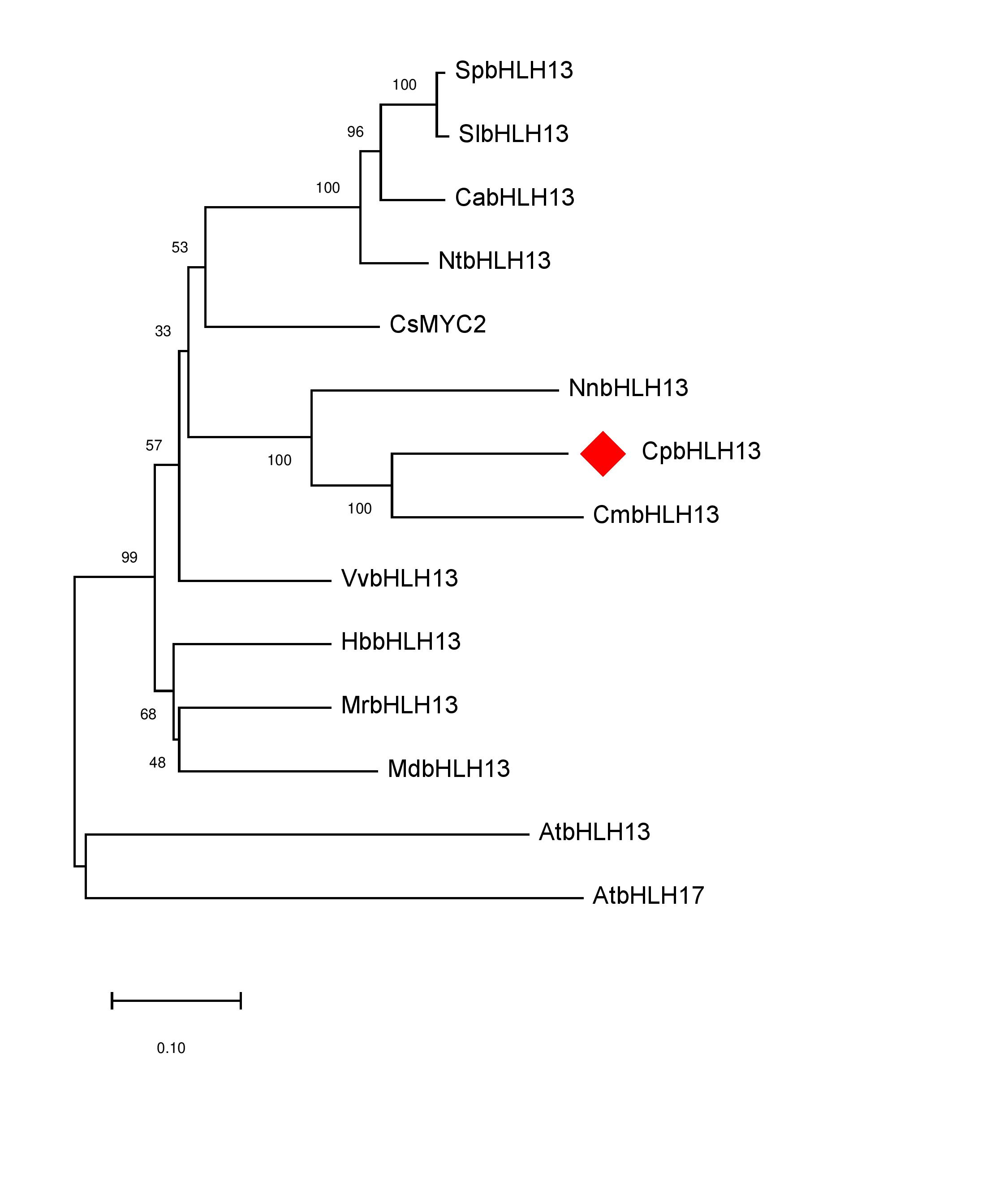

Supplement: Supplementary file 1 [file plants-09-00785-s001.zip › Supplementry data revised 21 June 20/Supplementary Final Figures 21 June/Supplementary Fig 6.jpg]

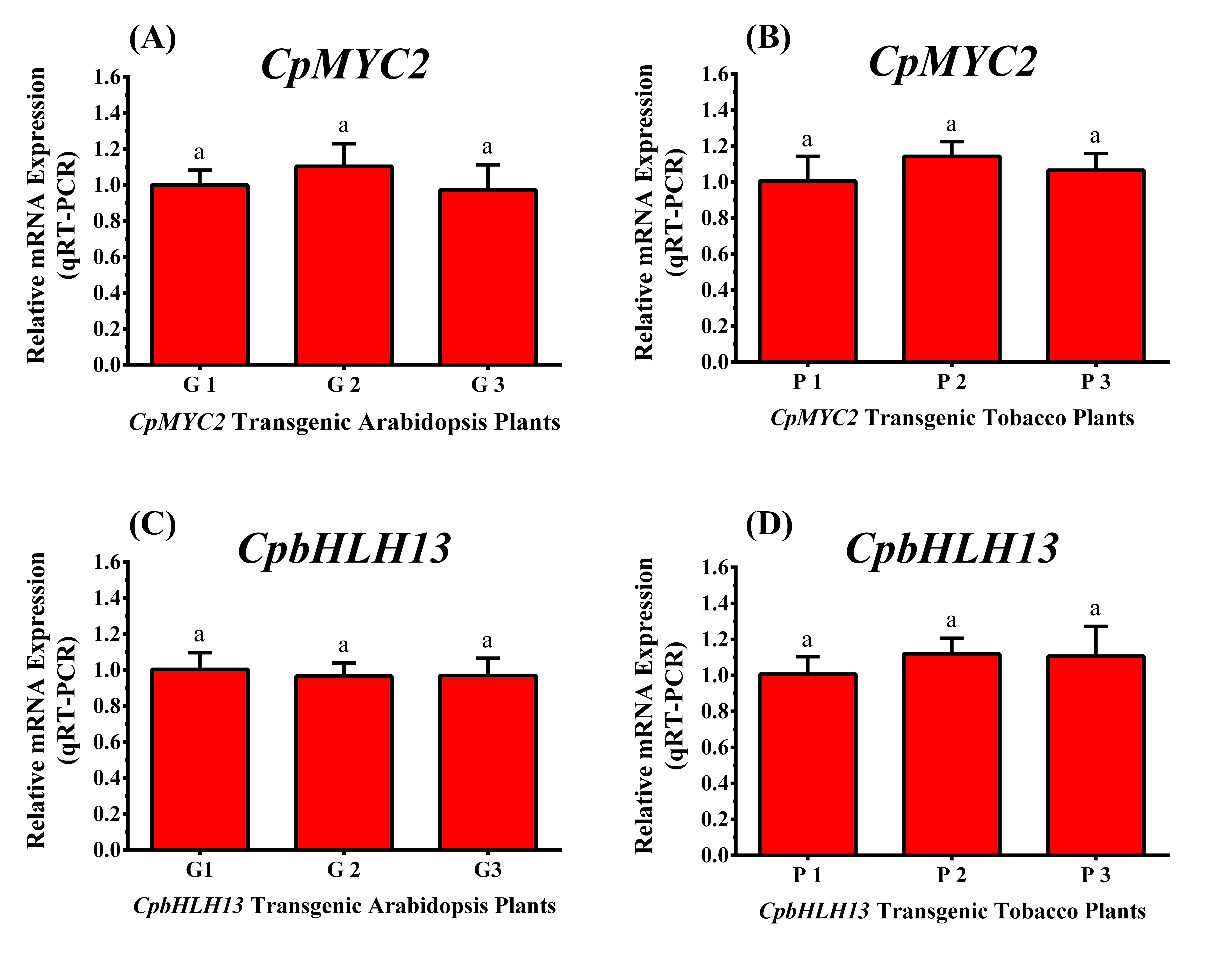

Supplement: Supplementary file 1 [file plants-09-00785-s001.zip › Supplementry data revised 21 June 20/Supplementary Final Figures 21 June/Supplementry Fig 7.tif]

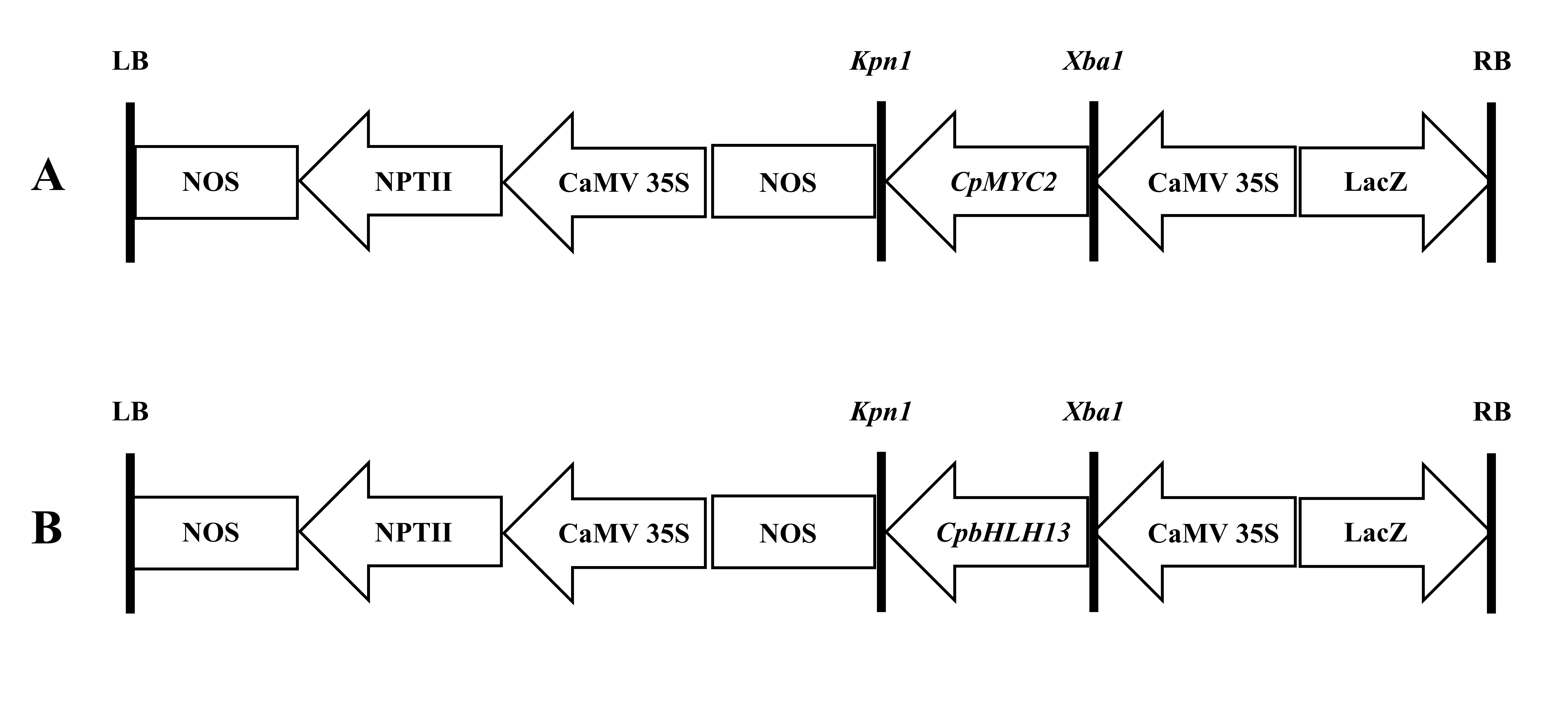

Supplement: Supplementary file 1 [file plants-09-00785-s001.zip › Supplementry data revised 21 June 20/Supplementary Final Figures 21 June/Supplementry Fig 8.tif]

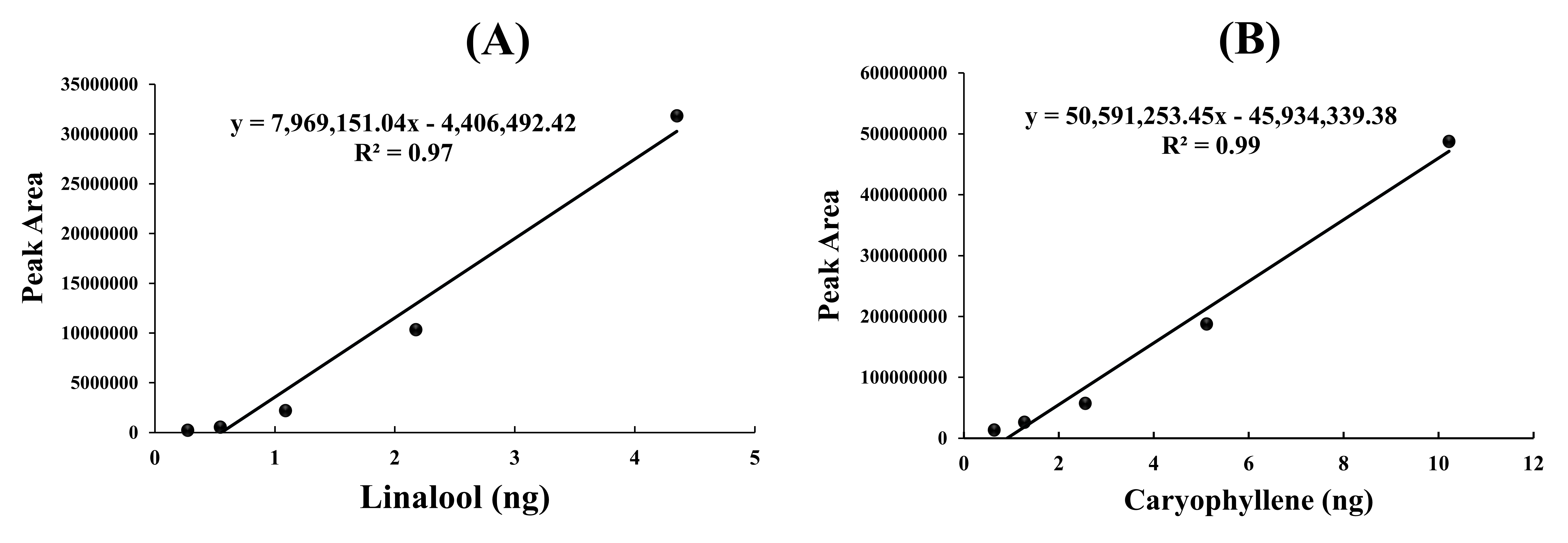

Supplement: Supplementary file 1 [file plants-09-00785-s001.zip › Supplementry data revised 21 June 20/Supplementary Final Figures 21 June/Supplementry Fig 9.tif]
